# An Optimization Framework for Data Collection in Software Defined Vehicular Networks

**DOI:** 10.3390/s23031600

**Published:** 2023-02-01

**Authors:** Patikiri Arachchige Don Shehan Nilmantha Wijesekara, Kalupahana Liyanage Kushan Sudheera, Gammana Guruge Nadeesha Sandamali, Peter Han Joo Chong

**Affiliations:** 1 Department of Electrical and Information Engineering, Faculty of Engineering, University of Ruhuna, Galle 80000, Sri Lanka; 2 Department of Electrical and Electronic Engineering, Auckland University of Technology, Auckland 1010, New Zealand

**Keywords:** vehicular network, data collection, optimization, SDVN

## Abstract

A Software Defined Vehicular Network (SDVN) is a new paradigm that enhances programmability and flexibility in Vehicular Adhoc Networks (VANETs). There exist different architectures for SDVNs based on the degree of control of the control plane. However, in vehicular communication literature, we find that there is no proper mechanism to collect data. Therefore, we propose a novel data collection methodology for the hybrid SDVN architecture by modeling it as an Integer Quadratic Programming (IQP) problem. The IQP model optimally selects broadcasting nodes and agent (unicasting) nodes from a given vehicular network instance with the objective of minimizing the number of agents, communication delay, communication cost, total payload, and total overhead. Due to the dynamic network topology, finding a new solution to the optimization is frequently required in order to avoid node isolation and redundant data transmission. Therefore, we propose a systematic way to collect data and make optimization decisions by inspecting the heterogeneous normalized network link entropy. The proposed optimization model for data collection for the hybrid SDVN architecture yields a 75.5% lower communication cost and 32.7% lower end-to-end latency in large vehicular networks compared to the data collection in the centralized SDVN architecture while collecting 99.9% of the data available in the vehicular network under optimized settings.

## 1. Introduction

A Vehicular Adhoc Network (VANET) is a key part of the Intelligent Transportation System (ITS), which necessarily consists of a wireless network of mobile vehicles. However, information dissemination in VANET is challenging due specifically to its characteristic of high mobility, frequent topology changes, and the presence of isolated nodes. VANET’s distributed approach finds it hard to address the above problem. A Software Defined Vehicular Network (SDVN) is a paradigm that has been introduced to improve the functionality of VANETs by enabling programmability and flexibility, which further facilitates the knowledge of the global view of the vehicular network. An SDVN applies a Software Defined Networking (SDN) concept in VANETs, where there exists a logically centralized control plane that collects data from the vehicles and roadside units to develop the global view of the vehicular network, which enables programmability and flexibility in vehicular networks.

There exist mainly three architectures for SDVNs: distributed, hybrid, and centralized, based on the degree of control of the control plane. Data collection is a fundamental requirement in SDVNs, as the controller takes network decisions based on the collected data. Furthermore, data collection is mandatory if machine learning is applied to identify patterns in the data and make improved network decisions accordingly. In the centralized architecture of SDVNs, data is first collected by the nodes and then transmitted to the centralized controller [1]. In the preceding method, all nodes first broadcast their data, and once broadcast data are received by the nodes, collected data will be transmitted to the centralized controller by each node. In traditional VANETs or, in other words, in a distributed SDVN architecture, nodes usually collect data using broadcasting and there is no communication with a centralized controller [2].

However, the problem is that there is no proper mechanism for data collection in a hybrid SDVN architecture (architecture that can vary the control on the nodes between fully centralized control to fully distributed control) found in the literature to the best of our knowledge. So far, data collection in a hybrid architecture has also occurred in the manner specified for a centralized architecture. However, we note that the data collection approach for a centralized architecture is not the ideal way to collect data. In such an approach, as all nodes act as both data unicasting agents and data broadcasting nodes, we observe that redundant data is collected at the controller. Due to redundant data transmission, there is an extra communication cost and resource utilization, which will also lead to higher latency despite its very high reliability. Thus, we observe that in a hybrid architecture, it is possible to effectively control data plane communication and control plane communication such that all data will be collected at the controller with minimum resource utilization, cost, and latency.

In this paper, we propose a novel data collection methodology for the hybrid architecture of SDVNs, where we use optimization techniques to effectively choose which nodes should broadcast and which nodes should unicast data subjected to the minimization of total communication cost, total communication delay, total communication overhead, etc. The data collection optimization framework is modeled as an integer quadratic programming problem where agents are selected to cover the whole vehicular network in order to transfer data from all nodes in the vehicular network to the controller. We identify two problems in the proposed optimization approach: node isolation and redundant data transmission if a new solution to the data collection optimization is not found when the vehicular network topology changes. As a solution for that, we propose to check for optimization frequently and make optimization decisions by inspecting the network link entropy change. For that, we formulate heterogeneous network link-state entropy by considering links with Road Side Units (RSUs) and vehicular nodes as two different types of links. We make the optimization decision by comparing the heterogeneous network link-state entropy change with a threshold value. Furthermore, we present a systematic way to collect both data and metadata (metadata are the data required to solve the optimization model, which consists of a timestamp, Node Identifier (ID), and a 1 hop Neighbor set IDs of each node in the vehicular network) in the initial data-gathering cycle and subsequent data-gathering cycles avoiding redundant data transmissions. In each subsequent data-gathering cycle, first, the broadcasting nodes should broadcast a status and neighbor ID set, while agent nodes should broadcast metadata during the same time step. Once the broadcasting is over, the agent nodes should unicast the collected data along with its own data to the controller, where the controller will extract metadata using the data unicast from agents to find a solution to the optimization problem. Finally, at the end of the cycle, the optimization solution will be broadcast to the nodes. Furthermore, we discuss the probable solutions of the proposed data collection optimization framework for a couple of vehicular network topologies. We further analyze the performance of the data collection of different SDVN architectures, including the proposed optimization framework, as a platform for future researchers to investigate.

As contributions to the existing literature, we present a novel optimization framework for data collection in the hybrid architecture of software-defined vehicular networks, which has a much lower communication cost, resource utilization, and end-to-end latency than existing data collection methodology for a centralized SDVN architecture. The proposed framework will be useful for making data-driven decisions using Machine Learning (ML)/Deep Learning (DL) in future software-defined vehicular networks. The limitation of the proposed method is that it is only applicable to the SDVNs, where both the data plane communication and control plane communication exist such that agent nodes and broadcasting nodes can be selected using optimization.

The rest of the paper is organized as follows. Section 2 reviews the existing literature providing a background for an SDVN and its existing architectures, Section 3 presents the proposed data collection mechanism in detail, Section 4 presents performance evaluation metrics, simulation environment configuration, and all the research experiments conducted with their results and analysis, Section 5 cross analyzes the results and compares them with the existing literature while discussing the results, and, finally, Section 6 concludes the paper and presents future work.

## 2. Background and Literature Review

A **Mobile Adhoc Network (MANET)** is an autonomous collection of mobile devices, such as smartphones, sensors, laptops, vehicles, etc., that communicate with each other over wireless links and cooperate with each other in a distributed manner to provide network functionality in the absence of fixed infrastructure [3]. These networks are characterized by self-organizing, dynamic, and volatile links between the wireless nodes and dynamic network topology [4]. Routing is challenging in MANETs due to the multi-hop network topology that can frequently change due to mobility [5]. Due to the mobility of the nodes, security vulnerabilities have arisen in MANETs such that attackers can eavesdrop, delete, modify, fabricate, and replicate protocol control packets or data packets more easily than wired networks [6]. Service and resource discovery are important aspects of MANETs, as they may have little or no knowledge of each other capabilities when there are no service or resource-discovery mechanisms [7]. As the mobile nodes of the MANET have limited energy, the energy efficiency of the network functions is very important [8].

A **vehicular adhoc network** is a subclass of MANET having no fixed infrastructure for communication and relying on vehicles themselves for network functionality [9]. A distinctive set of candidate applications such as collision warning and local traffic information for drivers, resources such as a licensed spectrum, rechargeable power source, and the environment having vehicular traffic flow patterns, privacy concerns, etc. distinctly identify VANETs from other MANETs [10]. Other distinct features which identify VANETs from MANETs are the high mobility of the nodes with dynamic network topology and the VANETs having an enormous number of nodes (vehicles) with high computational capacity [11]. VANETs are further characterized by the use of Dedicated Short Range Communication (DSRC), road-constrained mobility patterns, and not having significant power constraints [12]. A VANET works on the communication layer and plays a major role in future Intelligent Transportation Systems (ITS). In a VANET, each vehicle is identified as a node and equipped with an On-Board Unit (OBU), which is capable of exchanging information with other vehicles or Road Side Units (RSUs) [13]. RSUs are stationary communication nodes that are located near the roads in a VANET that enhances its connectivity [14]. Communication between vehicles is known as Vehicle to Vehicle (V2V) communication, while communication between vehicles and other infrastructure such as RSUs, tolls, internet access points, etc., is known as Vehicle to Infrastructure (V2I) communication [15]. Generally, V2X identifies the vehicle to everything communication. Sensors in vehicles and RSUs comprise a Vehicular Sensor Network (VSN) that can sense data related to traffic or environmental conditions, such as temperature, movement, pressure, vibration, etc., which are processed by vehicular applications and generate messages to be exchanged using the VANET [16]. Research and development in VANETs have dramatically increased due to the embracing of the IEEE 802.11p standard, which is a draft amendment to the IEEE 802.11 standard to add Wireless Access in Vehicular Environment (WAVE) for VANETs, and due to the licensed spectrum allocated for VANETs [17]. VANETs have applications in collision warning systems, optimal speed advising systems, driver assistance systems, entertainment, remote wireless diagnosis, etc. [18]. Privacy-preserving approaches are required for VANETs as the identifying information of vehicles, such as location, path, vehicle condition, etc., are exchanged in VANETs [19]. However, VANETs have their own set of challenges, such as high mobility of the nodes, security concerns, and routing issues [20].

**Software defined networking** is an emerging concept that enables network programming by promoting logical centralization of the network control, by separating the basic network control logic from switches and routers [21]. Traditional networking is hardware-based, where the control plane is distributed among network devices like routers. SDN involves the separation of the data plane and control plane compared to traditional network architecture, which encapsulates both data and control plane in the network devices [22]. SDN has three planes: an infrastructure plane, a control plane, and an application plane. Among numerous pros SDN brings in, flexibility and programmability stand out compared to conventional networks, as software can be produced via different vendors easily. In SDN, controlling is logically centralized, where a single protocol can be used to communicate between any number of hardware devices. SDN has greater visibility and also has the ability to define secure pathways compared to traditional hardware-based networking [23]. Using SDN, many new capabilities and services have been enabled, such as network virtualization and automation, orchestration for cloud services, traffic engineering, software engineering, etc. [24]. However, it has been noted that SDN faces challenges in terms of security, the inability of the centralized controller to single-handedly control all the traffic, the existence of only a few protocols to communicate between controller and applications, difficulty in integration with old networks that do not support OpenFlow protocol, etc. [25]. Another serious shortcoming of SDN is reliability, as the SDN controller tends to be a single point of failure [26].

Recently, researchers have applied SDN in a VANET, resulting in a new paradigm called **software defined vehicular network** enabling programmability and flexibility in vehicular networks [27]. Due to the inclusion of SDN in VANETs, many advantages are realized, including the improvement in routing, dynamic allocation of radio interfaces, and adaptive allocation of transmission power of the nodes due to network awareness of the SDN controller [28]. Further, due to the use of SDN, SDVN brings out an efficient network management mechanism for VANETs and due to the collection of network statistics, it improves networking applications such as routing, load balancing, and making global optimizations in VANETs. Further, it promotes network innovation, as new protocols for VANETs can be tested and deployed at a lower cost [29]. However, like any paradigm, SDVN also has its own set of challenges. The paper [30] discusses the security vulnerabilities that exist in each of the three planes of SDVNs. Further, network functions such as routing, and transmission control are challenging in SDVNs due to the high mobility of the nodes and dynamic network topology [31,32].

Based on the degree of control of the SDN controller, there are three architectures for SDVNs [33].

- **Centralized architecture**—This is the original architecture of an SDVN, where the control is logically centralized. In this approach, nodes in the data plane will execute actions according to the flow rules given by the SDN controller. This architecture has higher latency for the control plane communication between nodes and the centralized controller [34]. Further, there is a tendency for error when the control plane communication is lost or disrupted, and the scalability is low in this architecture.
- **Distributed architecture**—In this architecture, control is distributed among the end nodes where the nodes operate without any guidance from the centralized controller where the operation is similar to original VANETs. This architecture prevents the single point of failure and scalability issues found in the centralized control architecture. However, this architecture consumes more time than the centralized architecture to find the optimal routes [35].
- **Hybrid architecture**—This architecture has been proposed to overcome the limitations of both distributed control and centralized control architectures [36]. In this architecture, the centralized controller can vary the control over the nodes between full to zero based on the requirement such that it can behave as a mixture of centralized control and distributed control [37].

Another promising SDVN architecture is the Intelligent Digital Twin (IDT) based SDVN architecture having real-world SDVN and its intelligent virtual digital twin, which is a self-evolving virtual network using intelligent algorithms and decisions of the virtual network, is implemented in the physical network [38]. However, for this IDT-based SDVN architecture, road traffic prediction, the requirement of multi-source data fusion, energy consumption in constructing IDT, and running safety applications requiring low latency have been found to be challenging.

## 3. Materials and Methods

### 3.1. Overview of the Data Collection Mechanism

There exist data collection mechanisms for the centralized and distributed architectures of SDVNs as reviewed in the literature. In a centralized architecture, data is first broadcast to the neighbors, and then the collected data will be sent to the controller by all nodes, whereas in a distributed architecture, only the all-nodes broadcasting data step takes place. However, there is no data collection mechanism for the hybrid SDVN architecture to the best of our knowledge. In this paper, we optimize the data collection of the hybrid architecture of an SDVN.

Note that for the proposed data collection for a hybrid SDVN architecture, an **agent** is a node that collects data from neighbors and unicasts the collected data along with the node’s own data to the centralized controller. A **broadcasting node** is a node that broadcasts its own data to its neighbors.

The collected data can be either vehicle status or vehicular sensor measurements. The vehicle status is a collection of data comprising the Basic Safety Message (BSM), such as current time, position (latitude, longitude, elevation), transmission, speed, heading, steering wheel angle, acceleration, brake system state, and vehicle size [39]. In addition, vehicles can be employed with sensors such as Red Green Blue (RGB) cameras, depth cameras, Light Detection and Ranging (LIDAR) sensors, proximity sensors, Radio Detection and Ranging (RADAR) sensors, etc.

In subsequent data-gathering cycles of the proposed data collection framework for hybrid architecture, first, the broadcasting nodes will broadcast their data (status and/or sensor measurements) with the set of one-hop neighbors’ node ID for the broadcasting node ($S_{i}$), while the agent nodes will broadcast only metadata to their immediate neighbors. After broadcasting, the agent nodes need to send the collected data received from their neighbors, added with the agent node’s own data to the centralized server as the second step. Data is sent to the controller in order to make network decisions such as routing optimization, network management, load balancing, etc., based on the knowledge of the global network data. The data-collection process using the proposed optimization framework is described in detail in Section 3.3.2.

### 3.2. Formulating Factors Affecting Data Collection

Several factors governing the data collection process can be identified.

1. **Update/Data collection frequency (*f*)**—As vehicle status and sensor measurements are highly dynamic in a vehicular network, data have to be collected periodically at a sufficient frequency such that collected information depicts the current dynamics of the vehicle. If the frequency is too low, the knowledge generated regarding the vehicular network will be less valuable, as it has been generated for an older version of the vehicular environment. However, this frequency cannot be so high since it can cause an extra communication burden in terms of bandwidth and cost, and can result in congesting the communication channels. However, in such a case, the validity (accuracy) of the generated knowledge is higher. Therefore, this tradeoff has to be properly managed and optimized.
2. **Data content (L)**—The control plane must choose from the vehicle status and sensor measurement, and collect data which are only essential for knowledge generation. For *j^th^* node, we represent node’s status data payload size using $L_{st,j}$ and its sensor data payload size using $L_{se,j}$. Here, *p_j_* represents whether node *j* collects status data or not as given in Equation (1).
  (1) $$p_{j}=\left\{\begin{array}{c} 1;\;\;if\;node\;j\;collects\;status\;data\\ 0;\;\;otherwise \end{array}\right.$$
  Similarly, we use *q_j_* to represent the state of a collection of sensor data from the *j^th^* node as given in Equation (2).
  (2) $$q_{j}=\left\{\begin{array}{c} 1;\;\;if\;node\;j\;collects\;sensor\;data\\ 0;\;\;otherwise \end{array}\right.$$
  The collection of unwanted data can cost a waste of communication bandwidth and can exhaust the system. The decision to use whether the mined data from an OBU is directly sent to the control plane or not will depend on the SDVN architecture.
3. **The number of participating nodes as agents (*k*)**—One trivial way is to involve all the vehicular nodes in the data collection process where redundant information may be collected by the centralized control plane. So, such a scenario is not the optimum way to collect data as such a system will increase the vehicular network’s communication cost and overall latency of the system. In other words, all vehicles in the vehicular network may not be required to send or receive data. Consider a system that only collects vehicle status. In such a case, when there is a group of vehicles (a vehicle and its one-hop neighbors), the data can be collected from one agent of the vehicle group. This will reduce the communication overhead as the agent collects information broadcast from its one-hop neighbors and transmit it to the centralized controller along with its own data encapsulated in a single header. Furthermore, the number of participating agents is also SDVN architecture dependent. For a group of vehicles of the hybrid architecture, one agent needs to collect and send data to the centralized control plane. However, for the distributed architecture, there are no agents for data transmission, whereas, for the centralized architecture, all nodes act as agents.
4. **The delay (D)**—Some vehicular network applications such as safety (required latency < 100 ms) are delay sensitive [40]. The information should be collected such that the total delay does not exceed the required standards. In a multi-hop scenario, the total delay is the addition of delay at each hop. The total delay (D) in a single hop can be calculated as in Equation (3) [41].
  (3) $$\begin{array}{c} D=t_{trans}+t_{q}+t_{cont}+t_{proc}+t_{prop} \end{array}$$
  In Equation (3), $t_{trans}$ is the transmission delay, $t_{q}$ is the queuing delay, $t_{cont}$ is the contention delay, $t_{proc}$ is the processing delay, and $t_{prop}$ is the propagation delay. We calculate the delay corresponding to each term as in work [41]. Single-hop delay is involved in distributed SDVN architecture. Multi-hop delay is involved in both hybrid and centralized SDVN architectures.
5. **Cost associated with the communication channel (C)**—The communication channel used for data communication will depend on the SDVN architecture and the components involved in the communication. There are three candidates for the communication channel, namely: cellular, DSRC, and wired. Regardless of the architecture, we assume that data plane communication between Infrastructure and Infrastructure (I2I) (between RSUs, RSU, and control server, evolved node base station and control server, etc.) always uses wired communication [42]. Furthermore, regardless of architecture, V2V communication always uses the DSRC communication channel. However, V2I, and Infrastructure to Vehicle (I2V) communication will depend on the SDVN architecture. For example, in centralized and hybrid SDVN architectures, the control server is located at a remote place so each wireless node (OBU) has to use cellular communication to send data to the control server. However, in the distributed SDVN architecture, there is no cellular communication for V2I or I2V, and the local agent is responsible for collecting data for knowledge generation by exchanging data between the nodes using DSRC communication only. Here, we assume the communication cost as follows. We associated the least cost with the wired communication, a medium cost with the DSRC, and the highest communication cost with the cellular communication [43].
6. **Total overhead**—If each node of the vehicular network transmits data to the centralized server, each packet will have to be encapsulated with a header and transmitted. Alternatively, a data collecting agent can collect the broadcast packets received from its neighbors and transmit them together along with the agent’s data by encapsulating them with a header. The overhead will be less in the second scenario than when each node transmits data on its own to the central server. Therefore, when selecting agents, priority must be given to the nodes that contain a higher number of neighbors.

### 3.3. Proposed Data Collection Optimization Model

We derive the data collection problem as an Integer Quadratic Programming (IQP) model. Here, we have a network graph of G = (V,E), where V refers to the set of nodes (OBUs and RSUs), and E refers to the edges (connections) between the nodes. For the data collection problem, we use $x_{i}$ to represent the association of node *i* in the data collection process as a data collection agent, as given in Equation (4).

(4) $$x_{i}=\left\{\begin{array}{c} 1;\;\;if\;node\;i\;is\;an\;agent\;in\;the\;data\;collection\;process\\ 0;\;\;otherwise \end{array}\right.$$

For a given node *i*, we represent using $z_{i}$ the state of broadcasting of its data to immediate neighbors in the data collection process, as given in Equation (5). Note that the value of $z_{i}$ depends on the SDVN architecture since the decision to exchange data between neighbors depends on it.

(5) $$z_{i}=\left\{\begin{array}{c} 1;\;\;if\;node\;i\;is\;broadcasting\;data\;to\;its\;immediate\;neighbors\\ 0;\;\;otherwise \end{array}\right.$$

Furthermore, we use $S_{i}$ to represent the set containing all one-hop neighbors of a given node *i*. The total number of nodes in the system is represented using N and the total number of one-hop neighbors of a given node (*i*) is represented using $N_{i}$.

#### 3.3.1. Objective Function

The objective is to minimize the parameters listed below.

- The number of agents;
- Communication delay between nodes;
- Communication delay for agents;
- Communication cost per byte between nodes;
- Communication cost per byte for agents;
- Total payload of communication between nodes;
- Total overhead of communication for agents (maximize the number of neighbors of the agent nodes);

The above parameters can be mathematically written to formulate the objective function given in Equation (6). Each term in Equation (6) corresponds to the parameters given in the above list in the same order. Note that in the objective function given in Equation (6), $x_{i}$ and $z_{i}$ are the decision variables.

(6) $$\begin{array}{c} minimize\;\;\;C_{N}\sum_{i=1}^{N}x_{i}+C_{DN}\sum_{i=1}^{N}\left(z_{i}\sum_{m\in S_{i}}D_{im}\right)+C_{DA}\sum_{i=1}^{N}D_{i}x_{i}+C_{CN}\sum_{i=1}^{N}\left(z_{i}\sum_{m\in S_{i}}C_{im}\right)\\ +C_{CA}\sum_{i=1}^{N}C_{i}x_{i}+C_{LN}\sum_{i=1}^{N}N_{i}f\left(\left(p_{i}L_{st,i}\right)+\left(q_{i}L_{se,i}\right)\right)z_{i}-C_{O}\sum_{i=1}^{N}\left(x_{i}\sum_{m\in S_{i}}z_{m}\right) \end{array}$$

In Equation (6), $C_{N}$ is a large positive coefficient to stress the fact that the number of nodes participating in the data collection process should be minimized. N is the total number of nodes in the vehicular network. $k=\sum_{i=1}^{N}x_{i}$ is the total number of agent nodes participating in the data collection process. $C_{DN}$ is a positive coefficient for delay between nodes and $C_{DA}$ is the coefficient for delay of the agents. $C_{CN}$ is the cost coefficient for the communication between nodes, and $C_{CA}$ is the cost coefficient for the communication of agents, $C_{LN}$ is the coefficient for the overhead of the nodes, and $C_{O}$ is the coefficient for overhead. $D_{im}$ is the delay for communication from the $i^{th}$ node to its neighbor m. $D_{i}$ is the delay for communication from the $i^{th}$ agent to controller. Furthermore, $C_{im}$ is the communication cost associated with communication between the $i^{th}$ node and its $m^{th}$ neighbor. $C_{i}$ is the communication cost for transmitting collected data by the $i^{th}$ agent to controller. However, we can derive a simpler version of the objective function given in Equation (6) by reducing redundant terms and combining terms as follows. Note that the first term in Equation (6) minimizes the number of agents. However, when minimizing the communication delay of agents using the third term, and when minimizing the cost of agents using the fifth term in Equation (6), the total number of agents will be automatically minimized. Therefore, the first term of the objective function given in Equation (6) can be removed. Furthermore, we can observe similar terms for the communication cost per byte and communication delay (term 2 and term 4, term 3 and term 5 of Equation (6) are similar). Therefore, we define combined communication cost (B) as the encapsulation of communication delay and communication cost per byte for a given link. Accordingly, the combined communication cost from $i^{th}$ node to its $m^{th}$ neighbor ($B_{im}$) can be formulated as given in Equation (7).

(7) $$\begin{array}{c} B_{im}=\;C_{im}+D_{im} \end{array}$$

Similarly, the combined communication cost of an agent *i* ($B_{i}$) is the sum of the communication cost per byte and communication delay for the link that exists between the agent and the control server as shown in Equation (8).

(8) $$\begin{array}{c} B_{i}=\;C_{i}+D_{i} \end{array}$$

Furthermore, we can formulate the combined data packet ($L_{i}$) broadcast by node *i*, as given in Equation (9) by combining the status data and sensor data of the $i^{th}$ node.

(9) $$\begin{array}{c} L_{i}=\;p_{i}L_{st,i}+q_{i}L_{se,i} \end{array}$$

Therefore, by using Equations (6)–(9), we derive a simplified version for the objective function as shown in Equation (10).

(10) $$\begin{array}{c} minimize\;C_{CN}\sum_{i=1}^{N}\left(z_{i}\sum_{m\in S_{i}}B_{im}\right)+C_{CA}\sum_{i=1}^{N}B_{i}x_{i}+C_{LN}\sum_{i=1}^{N}N_{i}fL_{i}z_{i}-C_{O}\sum_{i=1}^{N}\left(x_{i}\sum_{m\in S_{i}}z_{m}\right) \end{array}$$

The objective function in Equation (10) is applicable to any data collection scenario. However, we can derive a more simplified version of the generic objective function given in Equation (10) for a special use case. Consider a scenario where only the status data is collected at a constant rate ($p_{i}=1,q_{i}=0,L_{i},f$ are constants). As the RSUs are stationary, their velocity and acceleration are zero, and the position is fixed. Thus, broadcasting status as I2I (from an RSU to another RSU) is not necessary. However, both vehicular nodes and RSUs can broadcast status in the DSRC communication channel (V2V, I2V, V2I). Thus, the combined communication cost for broadcasting from a given node *i* to its neighbor *m* ($B_{im}$) can be assumed to be constant as the communication cost and delay within the same communication channel (DSRC) is almost the same. Therefore, both the first and third terms in the generic objective function in Equation (10) can be reduced to one term and the objective function for this special case of status data collection can be written as in Equation (11). Our simulations will include only the status data collection, and thus they will be conducted only for the special case objective function derived in Equation (11). However, if sensor data is collected, the objective function in Equation (10) must be used, and as we simulate only status data collection, we use the objective function in Equation (11). However, it can be argued that solving the simplified case is almost equivalent to the generic case as terms 2 and 3 of Equation (11) are exactly the same as terms 2 and 4 of Equation (10), and the effect of term 1 of Equation (11) is equivalent to the effect of the combination of terms 1 and 3 of Equation (10), as term 1 of Equation (11) is formed by combining terms 1 and 3 of Equation (10).

(11) $$\begin{array}{c} minimize\;C_{CN}\sum_{i=1}^{N}N_{i}z_{i}+C_{CA}\sum_{i=1}^{N}B_{i}x_{i}-C_{O}\sum_{i=1}^{N}\left(x_{i}\sum_{m\in S_{i}}z_{m}\right) \end{array}$$

The objective function will be the same for any SDVN architecture. However, constraints will be different from SDVN architecture to architecture. Table 1 summarizes the notations used in the optimization model.

Some common facts regarding the optimization of data collection are given below.

#### 3.3.2. Common Facts about Optimization of Data Collection

**Figure 1:**
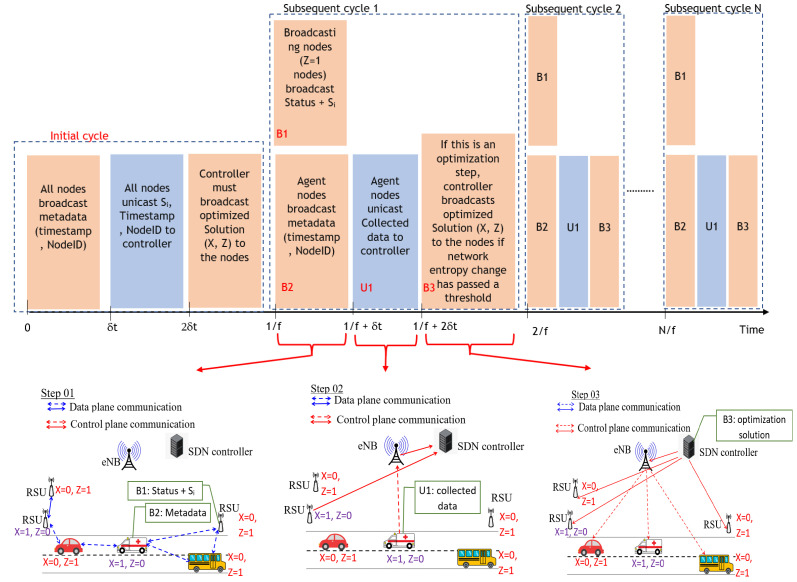
The process of status data, metadata, and optimization solution transferring in each data gathering cycle.

The process of status data, metadata, and optimization solution transferring in each data-gathering cycle is graphically shown in Figure 1.

**Figure 2:**
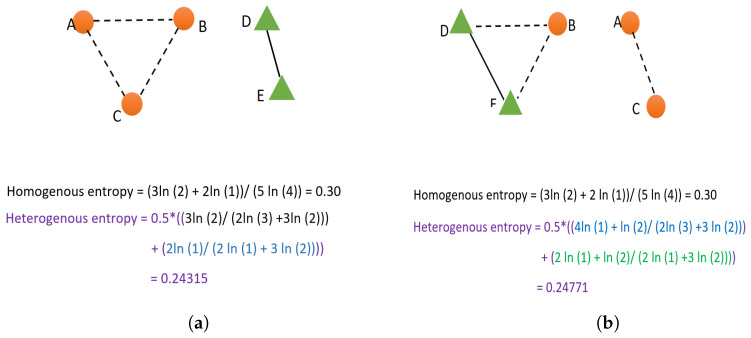
Entropy calculation for two network instances. (**a**) Vehicular network instance at time t−1; (**b**) Vehicular network instance at time t.

- Before the data collection is optimized for the very first time, all nodes should broadcast their timestamp and node ID (**broadcasting metadata**), as shown in Figure 1. This step is done to identify all one-hop neighbors’ metadata, such as the total number of neighbors ($N_{i}$), node ID set of one-hop neighbors ($S_{i}$) for the very first time which is required to solve the optimization problem. Once the data collection is optimized (after an optimization solution is found), using the broadcasting neighbors around a given node, a part of this metadata can be collected using the timestamp and node ID information which are already included in the data as shown in block B1 in Figure 1. However, there can be neighbors of an agent node who do not broadcast data to that agent node (when the neighbor node is another agent). Therefore, once the data collection is optimized, metadata must only be broadcast from the agent nodes, since metadata can be extracted using the data broadcasting nodes ($z_{i}=1$). In such nodes ($z_{i}=1$), we embed $S_{i}$ along with the status data in the broadcasting data packet as shown in block B2 in Figure 1. Metadata broadcasting of the agents should occur at a frequency of *f*, where *f* is the data collection frequency. There is another parameter called the nominal optimization frequency ($f^{\prime }$) where the *f* and $f^{\prime }$ are synced by the relationship given in Equation (12). In Equation (12), *k* is a positive integer such that the data collection frequency (*f*) should be a multiple of nominal optimization frequency ($f^{\prime }$). We call this the nominal optimization frequency since the decision to optimize or not is decided based on the entropy change of the network. At each step of nominal optimization frequency ($f^{\prime }$), entropy change will be inspected and the decision to optimize or not will be made, except for the first data-gathering cycle, which optimization solution must necessarily be found regardless of network entropy;
  (12) $$\begin{array}{c} f=\;kf^{\prime } \end{array}$$
- Before the data collection is optimized for the very first time, all nodes should unicast the node ID set of all one-hop neighbors ($S_{i}$) along with the sender’s node ID, and timestamp values to the controller as shown in Figure 1. We can call it **unicasting uplink metadata** for easy reference. Using these data, $B_{i}$ for each node can be computed at the controller. However, once a solution to the optimization problem is found, using the uplink data transmitted from the agents, a set of optimization parameters for all nodes can be computed. Therefore, the separate unicasting of metadata for the nodes is not required except for the first data-gathering cycle. Furthermore, unicasting the metadata $S_{i}$ of a given node that has not changed compared to the previous time step, can be omitted from having to be sent to the controller to reduce the metadata sent to the controller. For instance, if the topology around the neighborhood of a given node has not changed, then $N_{i},S_{i}$ values will be constants. However, if the topology has been changed around a neighborhood of a given node, only such nodes $S_{i}$ should be embedded with the data and sent to the controller. Thus, at a given instant of time, agent nodes will unicast collected status data from the neighbors with its own data along with a set of $S_{i}$, which the topology around their neighborhood has changed, thus reducing the communication burden, as shown in block U1 in Figure 1;
- The optimization problem for data collection is solved by the controller (control plane). For this purpose, the controller should collect all $S_{i}$ values from the nodes and compute $N_{i}$ accordingly, which is the size of set $S_{i}$. $B_{i}$ can be computed at the controller, by calculating $C_{i}$ based on the communication channel type and by calculating $D_{i}$ using the timestamp information received from unicasting uplink metadata or using the data itself of the agent nodes in order to solve the objective given in Equation (11);
- Note that the solution of the optimization problem is valid for a given instance of time, when the $S_{i}$, $B_{i}$, and $N_{i}$ are constants for that particular instance of time. However, these coefficients are time-varying as the topology of the vehicular network changes due to high mobility. The decision to optimize or not is taken by examining the change in entropy of the present network compared to the last optimized network. The normalized link entropy (*H*) of a homogeneous network [44] can be calculated as given in Equation (13);
  (13) $$\begin{array}{c} Homogeneous\;Normalized\;Entropy\;\left(H_{hom}\right)=\;\frac{\sum_{i=1}^{N}ln\;\left(N_{i}\right)}{N*ln(N-1)}\;;N_{i}\neq 0 \end{array}$$
- In Equation (13), *N* represents the total number of nodes in the network, $N_{i}$ represents the degree of node *i* or in other words, the total number of neighbors of the $i^{th}$ node. Homogeneous normalized link entropy ($H_{hom}$) has a value in the interval [0, 1]. Isolated nodes having zero degrees ($N_{i}=0$) should not be substituted for $N_{i}$ in Equation (13), however, those nodes are considered in the total number of nodes (*N*). However, $H_{hom}$ is defined for a homogeneous network. The vehicular network is heterogeneous, as there exist mainly two types of nodes: vehicular nodes and RSUs. The optimization parameters also differ among RSUs and vehicular nodes. If we identify RSUs and vehicles as homogeneous nodes and calculate normalized network entropy (*H*), there can be instances where the *H* does not change (degree/number of neighbors has not changed), but the neighborhood of nodes has changed with respect to communication links. For example, consider a network having three vehicular nodes and two RSU nodes, as shown in Figure 2. If we consider the network as homogeneous with respect to nodes, both network instances in Figure 2 will have the same homogeneous entropy ($H_{hom}$). However, the topology of the network has clearly changed, as evident from Figure 2. Thus, we calculate the normalized homogeneous link entropy with respect to vehicular nodes and RSU nodes separately and obtain the average of those two to compute the heterogeneous network entropy ($H_{het}$) as given in Equation (14). In Equation (14), $N_{v}$ is the total vehicle nodes, $N_{r}$ is the total RSU nodes, $N_{iv}$ is the total vehicular nodes in the neighborhood of a node *i*, $N_{ir}$ is the total RSU nodes in the neighborhood of a node *i*. As evident from the sample calculation for two network instances in Figure 2, the $H_{het}$ is different for two instances of the network;

(14) $$\begin{array}{c} H_{het}=\;0.5*\left(\frac{\sum_{i=1}^{N}ln\;\left(N_{iv}\right)}{N_{r}*ln\left(N_{v}\right)+N_{v}*ln\left(N_{v}-1\right)}+\frac{\sum_{i=1}^{N}ln\;\left(N_{ir}\right)}{N_{r}*ln\left(N_{r}-1\right)+N_{v}*ln\left(N_{r}\right)}\right)\\ \;;N_{iv},N_{ir}\neq 0 \end{array}$$

- When the network topology changes, if a new solution to the proposed data collection is not found, we can identify two problems, as given below.
  − Not receiving data from broadcasting nodes which have become isolated or gone out from a neighborhood of an agent;
  − Redundant data collection from agents, as agents have moved to the neighborhood of other agents;

**Figure 3:**
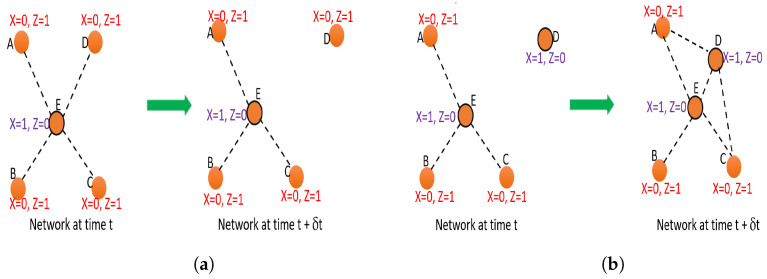
Demonstration of problems that arise if data collection is not optimized when the network topology changes; (**a**) Network instances showing data loss of broadcasting nodes due to isolation; (**b**) Network instances showing redundant data collection by agent nodes due to an agent moving to the neighborhood of another agent.

These two problems are graphically depicted in Figure 3. As evident from Figure 3a, node D, which was a broadcasting node at time *t*, has become isolated at time t+δt. As it has been assigned as a broadcasting node by the optimizer in the previous time step, in a new network instance when it is isolated from the rest of the network, node D’s data will not be collected by any agent, and will not be received to the control plane. However, if a new optimization solution is found at t+δt, node D will be assigned as an agent. Thus, when the topology changes, data loss can occur.

Consider the redundant data transmission problem depicted in Figure 3b. At time *t*, node D is assigned as an agent by the optimizer as it is an isolated node. At time t+δ, node D has moved close to nodes A, E, and C such that those nodes have become node D’s neighbors. However, as node D is assigned as an agent, node D will collect data from nodes A and C as well and send it to the control plane, while node E also collects data from nodes A and C and sends it to the control plane. However, if a new solution to the optimization problem is found at time t+δt, this problem can be avoided as the optimizer will convert node D into a broadcasting node and keep node E as the only agent. However, performing optimization for the slightest change in network entropy can cause a relatively higher channel utilization and cost, as will be proven in Section 4.3.1. Even though the best approach to avoid the above two problems is to optimize for even the slightest change in entropy (or without considering an entropy threshold at all), in order to save additional resources that are utilized additionally for an optimization step like downlink broadcasting, we can perform the optimization if and only if the heterogeneous normalized link entropy ($H_{het}$) of the network changes significantly compared to the last optimized network state. However, as pointed out before, there can be a loss of packets if broadcasting nodes become isolated and a new solution to the optimization problem for data collection is not found for the new time step. Thus, the entropy threshold must be selected by inspecting the Packet Delivery Ratio (PDR) such that the entropy threshold is not too high such that packet losses will occur. Thus, there is a trade-off between packet delivery ratio (reliability) and communication resource utilization when deciding the entropy threshold value. We compare the absolute value of the entropy change with a threshold (T) to decide on whether to optimize or not. The value of T can be set in the range [0, 1], and we decide on the optimum value of T experimentally;

- Once the solution for the optimization of data collection is computed ($z_{i},x_{i}$ are found using the optimization model), the solution should be broadcast back to all nodes, as shown in block B3 in Figure 1. These data can be called **broadcasting downlink metadata**;
- The transmission period (the reciprocal of data transmission frequency) should be less than or equal to the maximum time period allowed for an update ($T_{max}$). This constraint is given in Equation (15). This time period depends on the type of data collected according to the 3rd Generation Partnership Project (3GPP) standard;

(15) $$\begin{array}{c} \frac{1}{f}\leq \;T_{max} \end{array}$$

#### 3.3.3. Data Collection Method for Hybrid SDVN Architecture

The highest level of optimization for the data collection is possible in the hybrid SDVN architecture. Constraints specific to this hybrid SDVN architecture are given in the following section.

**Specific Constraints**

SDVN nodes (OBUs and RSUs) share their data with neighbors, and then collected data are sent to the control server using cellular or wired communication. In a hybrid SDVN architecture, all nodes do not need to participate in the data collection as agents, and all nodes do not need to broadcast data to neighbors as well. If all nodes participate in both types of communication, redundant data will be transmitted, as data has already been exchanged between the nodes. The minimum number of agents should be selected such that those agents cover the whole vehicular network. Any given node must be either an agent or should have at least one agent in its neighborhood in order for the agents to cover the whole vehicular network. This constraint is given in Equation (16).

(16) $$\begin{array}{c} x_{i}+\sum_{m\in S_{i}}x_{m}\;>=\;1;\;\forall \;i\in S \end{array}$$

In Equation (16), S is the set of one-hop neighbors of the whole vehicular network. If a node is selected as an agent, it does not need to broadcast to its neighbors, and if a node is selected as a broadcasting node, that node does not need to be an agent, as shown in the constraint given in Equation (17). In other words, every node in the network is either an agent or a broadcasting node.

(17) $$\begin{array}{c} z_{i}\;+\;x_{i}\;=1;\;\forall \;i\in S \end{array}$$

The total payload transmitted by the agents per unit of time after discounting the same data transmitted by multiple agents should be equal to the addition of data contained in each node. This constraint is given in Equation (18). When a given node broadcasts to multiple agents, such data should be counted only once. The negative term in Equation (18) ensures that such nodes are counted only once. The *max* function in Equation (18) returns the maximum value of the two inputs given to that function.

(18) $$\begin{array}{c} \sum_{j=1}^{N}L_{j}\;=\;\sum_{i=1}^{N}x_{i}\left(L_{i}+\sum_{l\in S_{i}}L_{l}z_{l}\right)-\sum_{i=1}^{N}L_{i}z_{i}max\left(\left(\left(\sum_{m\in S_{i}}x_{m}\right)-1\right),0\right) \end{array}$$

However, by re-inspection, it can be found that when all agents cover the whole network, the agents will automatically transmit the total payload available in nodes of the network. Therefore, out of constraint Equations (16) and (18), only one is necessary and sufficient. Thus, we can remove quadratic constraint Equation (18) and keep linear constraint Equation (16). Thus, for the hybrid architecture, the data collection can be optimized by selecting the minimum number of agents satisfying the constraints given in Equations (16) and (17) for the objective function given in Equation (10) or for the simplified objective function in Equation (11) for the special case of collecting status data.

#### 3.3.4. Data Collection in Centralized and Distributed SDVN Architectures

In a centralized architecture, data is exchanged between the nodes first, before sending to the control server ($z_{i}=1;\;\forall \;i$). All nodes need to participate in the data collection as agents ($x_{i}=1;\;\forall \;i$)

In a distributed architecture, all data are exchanged between the nodes ($z_{i}=1;\;\forall \;i$) and none of the nodes need to participate in the data collection as agents ($x_{i}=0;\;\forall \;i$).

Therefore, the decision variables ($x_{i}$ and $z_{i}$) for the optimization of data collection are bound to fixed values for all the nodes in both centralized and distributed architectures. Hence, the data collection does not need to be optimized using the proposed optimization framework, as there exists only one solution to the IQP problem.

### 3.4. Sample Solutions to the Optimization of Data Collection

**Figure 4:**
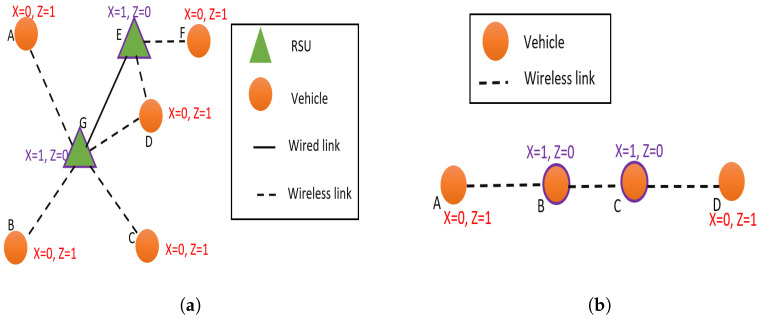
Network topologies showing the solution of the optimizer for data collection in Hybrid architecture. (**a**) Ad hoc topology; (**b**) Chain topology.

Let us predict the solution of the optimizer for several simple network topologies. Consider the network topologies given in Figure 4.

When considering the ad hoc topology given in Figure 4a, two RSU nodes E and G, will be selected as agents. Agents E and G cover the whole vehicular network (A–G), thus satisfying the constraint in Equation (16). The selection of node G as an agent is obvious as it has four neighbors (A,B,C,D) broadcasting to it. According to the third term of the objective function derived in Equation (10), an agent should have a higher number of neighbors broadcasting to it. In addition, agents should be selected such that the combined cost of an agent is minimum according to the second term of the objective function given in Equation (10). RSUs will have lower $B_{i}$ value due to low communication cost and low delay in Ethernet links. Hence, RSU E will be selected as the agent which has two broadcasting neighbors and a lower $B_{i}$ value compared to vehicle F, which has only one neighbor and has a higher $B_{i}$ value.

In order to gain more insight into the working of the optimization for data collection we can inspect the solution for the chain topology given in Figure 4b. In order to satisfy the constraints, it should be noted that two agents and two broadcasting nodes should be selected for the chain topology. The effect of the agent’s cost (second term of the objective function in Equation (10)) is the same for any selection of the node. The choice of agents and broadcasting nodes depends on the minimization of payload exchanged between the neighbors and the maximization of the payload transmitted to the agents. It should be noted that when the two middle nodes (B and C) are selected as agents, each node B and C will have one broadcasting neighbor. Thus, the total payload broadcast will be limited to two packets. For any other choice of agents (other than both B and C are selected as agents), the total broadcast packets will be greater than two. Thus, the optimizer will select B and C as agents and set A and D as broadcasting nodes.

## 4. Results

### 4.1. Performance Evaluation Metrics

We evaluate the performance of the optimization model for the hybrid architecture’s data collection using the following evaluation metrics.

#### 4.1.1. Average Communication Cost ($\bar{C}$)

The average total communication cost per node per data-gathering cycle can be computed by calculating the mean of the total communication cost of all the nodes for all data-gathering cycles, as given in Equation (19). The lower value of $\bar{C}$ indicates that the data collection method induces a lesser communication cost on average.

(19) $$\begin{array}{c} \bar{C}\;=\frac{1}{KN}\sum_{m=1}^{K}\sum_{i=1}^{N}\sum_{j=1}^{L}C_{j}P_{ij} \end{array}$$

In Equation (19), K is the total number of data-gathering cycles, and *L* is the total number of communication channels used by the $i^{th}$ node. $C_{j}$ represents the communication cost per byte for communication using the $j^{th}$ communication channel whereas $P_{ij}$ is the total packet size for sending a packet from the $i^{th}$ node using the $j^{th}$ communication channel.

#### 4.1.2. Average Channel Utilization (${\bar{U}}_{j}$)

The average channel utilization for $j^{th}$ communication channel (${\bar{U}}_{j}$) can be calculated as given in Equation (20). The channel utilization measures the degree of occupancy of a given channel for communication.

(20) $$\begin{array}{c} {\bar{U}}_{j}\;=\frac{100}{K}\sum_{m=1}^{K}\frac{total\;channel\;utilization\;time\;per\;data\;cycle}{total\;time\;of\;the\;data\;cycle} \end{array}$$

A high score for channel utilization (${\bar{U}}_{j}$) indicates that the channel is frequently utilized in the network. A lower channel utilization value is desirable.

#### 4.1.3. Average End-to-End Latency ($\bar{T}$)

The average end-to-end latency per node per data-gathering cycle ($\bar{T}$) can be calculated as given in Equation (21). Latency is a measure of delay taken to send a packet from a source to a destination. In centralized and hybrid architectures of SDVNs, data packets are originated in the nodes (OBUs or RSUs), and are sent to the control server node. A low latency value is desirable.

(21) $$\begin{array}{c} \bar{T}\;=\frac{1}{KN}\sum_{m=1}^{K}\sum_{i=1}^{N}\left(T_{ri}-T_{si}\right) \end{array}$$

In Equation (21), $T_{ri}$ is the receiving timestamp of the packet transmitted by the $i^{th}$ node at the destination, whereas $T_{si}$ is the sending timestamp of the packet by the $i^{th}$ source node. Usually, end-to-end delay is calculated among data packets transmitted from one vehicle to another (between two data plane units). However, the endpoints for calculating latency in this research are different, and packets consist of metadata.

#### 4.1.4. Optimization Percentage ($\bar{P}$)

We define optimization percentage ($\bar{P}$) as the proportion of times which the optimizer was called with respect to the total times the entropy threshold was checked. It can be calculated as given in Equation (22).

(22) $$\begin{array}{c} \bar{P}\;=\frac{Total\;times\;optimizer\;was\;called}{Total\;times\;entropy\;threshold\;was\;inspected} \end{array}$$

A high score for $\bar{P}$ indicates that the entropy change of the network has been higher than the entropy threshold most of the time.

#### 4.1.5. Average Packet Delivery Ratio ($\bar{R}$)

The average packet delivery ratio per data-gathering cycle ($\bar{R}$) measures the reliability of a system as it measures the proportion of packets that were delivered out from the total packets sent. Equation (23) can be used to calculate the packet delivery ratio. The packet source is either a vehicular node or an RSU node.

(23) $$\begin{array}{c} \bar{R}\;=\frac{100}{K}\sum_{m=1}^{K}\frac{Total\;packets\;received\;at\;destination}{Total\;packets\;sent\;from\;the\;source} \end{array}$$

The destination is the controller for hybrid and centralized SDVN architectures, while the destination is another vehicular or RSU node for the distributed architecture. Reliability refers to the maximum tolerable packet loss rate at the application layer [45]. An $\bar{R}$ value close to 100 is desirable.

The PDR in centralized and hybrid SDVN implies how much the controller is aware of the underlying data plane. However, in a VANET (distributed SDVN), the meaning of the PDR is how much information is exchanged between the neighbors. This information collection (in a centralized and hybrid SDVN) and exchange (in a VANET) lays the foundation for improved network functions such as routing.

### 4.2. Configuration of the Simulation Environment

We simulate the vehicular network in Network Simulator 3 (NS3) version 3.35, which has a higher realism in the models, uses C++ programming language, is architected similarly to Linux computers, and has internal interfaces and application interfaces [46].

#### 4.2.1. Configuration of Vehicular Mobility Scenarios

**Figure 5:**
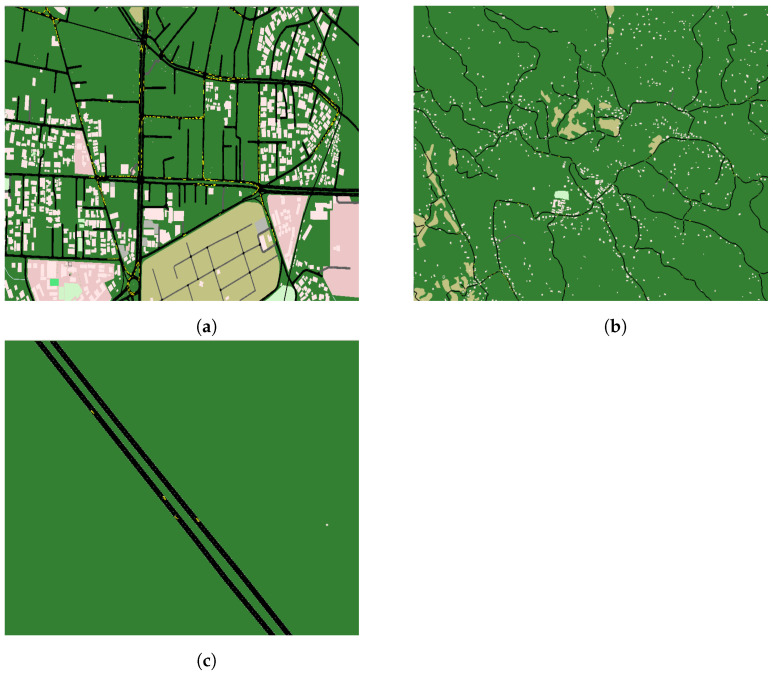
Different vehicular network mobility scenarios. (**a**) Vehicular network in an urban area in Colombo; (**b**) Vehicular network in a non-urban area in Neluwa; (**c**) Vehicular network on the southern expressway in Dodangoda.

The communication requirement of vehicular networks varies depending on the mobility scenario. Using the OpenStreetMap utility, we chose areas in Sri Lanka with maps with a size of 1500 m × 1500 m in the municipal town of Colombo, 6000 m × 6000 m in the Neluwa area, and an area the size of 4000 m × 7500 m along the Southern expressway in the Dodangoda area to represent urban, non-urban, and autobahn vehicular traffic, respectively, as shown in Figure 5.

Mobility traces for each scenario are generated using Simulation of Urban Mobility (SUMO) and OpenStreetMap. We configure the SUMO configuration file to add 200 vehicles (60 passenger cars, 60 trucks, 60 buses, and 20 motorcycles) for urban and non-urban scenarios, and 200 passenger cars for the autobahn mobility scenario, and generate mobility traces for each case by varying the maxSpeed parameter of all the vehicle types in increments of 10 kph (2.78 ms^−1^). The SUMO utility selects the maximum speed of a vehicle as the minimum of the maxSpeed parameter of the vehicle category and the speed limit of the particular lane. By increasing the maxSpeed parameter, we expect the average mobility to increase. However, the speed is not only constrained by the maximum allowable speed since many other factors, such as road signs and rules which the vehicle has to obey, the presence of other vehicles, intersections, obstacles, etc., governing the speed of a vehicle exist. In the Colombo municipal area (urban town), as shown in Figure 5a, most of the road speed limits are around 40–60 kph. Therefore, we vary the maximum speed parameter of the vehicles from 0–60 kph. However, for the Gelanigama expressway (autobahn scenario), we set the speed limit of the expressway to 250 kph to simulate high-speed mobility scenarios, thus we vary the maxSpeed parameter of the vehicles in the autobahn scenario from 0–250 kph. In the non-urban area of Neluwa, the speed limit of the roads is set to 100 kph such that the maxSpeed parameter of the vehicles is changed from 0–100 kph. Further, by modifying the SUMO configuration file, we set trips of the 200 vehicles to start at time t=0, set the maximum number of vehicles in the simulation for 200, and set the simulation end time as 600 s. Furthermore, to prevent the vehicles from leaving the map during the course of the simulation, we reroute a given vehicle back to the source edge, upon reaching the destination edge of the map and vice versa. We had to set the maximum number of vehicles to 200, as simulations were run in a computer with eleventh generation Intel Core i7 11800H CPU with 16 GB RAM, where further increments of the number of nodes resulted in very long simulation times. Therefore, due to limited processing and memory resources, we could not simulate the high traffic density scenario of 3000–4000 vehicles per square kilometer. However, we have been able to represent a fairly moderate traffic density scenario, since we have spawned 200 vehicles in the given map. Using the SUMO configuration file and the trip files, the Tool Command Language (TCL) file is generated for the mobility scenario, which can be imported to the NS3 simulation, which represents real-world vehicular traffic. The number of vehicular nodes (*N*) can be varied in the NS3 simulation to any number less than 200 based on the requirement where the particular number of vehicular nodes (say X) will extract mobility traces from the generated TCL file for the first X nodes automatically.

#### 4.2.2. Configuration of DSRC

We configure the Wi-Fi class available in NS3 using DSRC parameters. First, we set the Wi-Fi standard as IEEE 802.11p and set the remote station as a constant rate Wi-Fi manager. We set the Request to Send–Clear to Send (RTS–CTS) threshold of 65,535 bytes in order to disable the RTS–CTS flow control mechanism, as DSRC transmission is limited to one-hop broadcasts in this research. Furthermore, we set the maximum retransmission attempts for packets smaller than the RTS–CTS threshold to seven and that for packets larger than RTS–CTS threshold to four. Retransmission attempts are set to a higher value (seven as the packet size is always less than the RTS–CTS threshold) in order to increase the reliability of DSRC communication. According to the Federal Communication Commission (FCC), the maximum allowable Equivalent Isotropic Radiated Power (EIRP) at 5.9 GHz band is 44 dBm [47]. We consider the transmission powers 33 dBm, 41 dBm, and 44 dBm to satisfy the minimum coverage requirements of 139 m, 278 m, and 347 m for urban, non-urban, and autobahn vehicular environments, respectively. The preceding coverage has been standardized considering the maximum relative speed in each of the mobility scenarios [48]. A minimum throughput of 10 Mbps is required if only status information such as safety-related driving maneuvers are collected [48]. As our simulations involve the collection of such data, we choose the physical layer Orthogonal Frequency Division Multiplexing (OFDM) data rate of 12 Mbps to satisfy the throughput requirement. The propagation loss model of the DSRC channel is set as Cost-Hata (Cost 231) for the urban scenario and as the “Three Log Distance Propagation Loss” model for suburban and autobahn scenarios. Both Cost231 and Log Distance are deterministic path loss models. Cost 231 has been used to model path loss in urban scenarios in DSRC [49]. The three log distance propagation loss model assumes an exponential path loss over the distance, which is designed for suburban scenarios having three exponents for near, middle, and far fields [50]. An error rate model mark packets as errors based on a predetermined method. We set the error rate model as the “Nist Error rate model,” which models different error rates for different OFDM modulations in IEEE 802.11 [51].

#### 4.2.3. Configuration of Long Term Evolution (LTE) Standard

We use Cost-Hata (Cost 231) as the path loss model of LTE for all urban, non-urban, and highway scenarios, as it has been extensively used in Europe and North America to model transmission loss in cellular communication [52]. We enable the uplink power control in LTE, which is a technique to mitigate interference at the same time while maximizing the power of the desired received signal [53]. The maximum transmit power is set at 23 dBm following the 3GPP standard [54]. The Radio Resource Control (RRC) which is the signaling exchanged between the user equipment and the Evolved Node Base station (eNodeB), is enabled, and error models for control and data channels are also enabled. The Bit Error Rate (BER) for “PiroEW2010” Adaptive Modulation and Coding (AMC) scheme is set as 0.00005 [55]. Furthermore, we dynamically set the Sounding Reference Signal (*SRS*) periodicity based on the number of user equipment (*N*) as given by Equation (24).

(24) $$\begin{array}{c} SRS\;periodicity=\left\{\begin{array}{cc} 2\; & ;\;if\;0\;\leq \;N\;\leq \;1\\ 5\; & ;\;if\;2\;\leq \;N\;\leq \;4\\ 10\; & ;\;if\;5\;\leq \;N\;\leq \;9\\ 20\; & ;\;if\;10\;\leq \;N\;\leq \;19\\ 40\; & ;\;if\;20\;\leq \;N\;\leq \;39\\ 80\; & ;\;if\;40\;\leq \;N\;\leq \;79\\ 160\; & ;\;if\;80\;\leq \;N\;\leq \;159\\ 320\; & ;\;if\;160\;\leq \;N \end{array}\right. \end{array}$$

In Equation (24), $N=N_{vehicles}+3$, which consists of all vehicle nodes, a Packet Data Network (PDN) Gateway (PGW), Serving Gateway (SGW), and a controller node equipped with LTE network devices. The fading model, which models the multi-path propagation effects in LTE used in uplink and downlink, is set as the “Trace Fading Loss Model”. As the user equipment represents vehicles having high mobility, the fading trace for 60 kph is used for the simulation. We set the trace length to 10 s, the number of samples in the trace to 10,000, and the trace window size to 0.5 s. Furthermore, an Evolved Packet Core (EPC) network for the functioning of the LTE is implemented by installing PGW node and SGW using a point-to-point EPC helper with a data rate of 1000 Mbps and a delay of 10 μs. In order to have a fair comparison between different simulation runs, we place the eNodeBs at fixed positions in a simulation run and place PGW and SGW nodes in the neighborhood of the eNodeBs as it can occur in a real world scenario.

#### 4.2.4. Configuration of RSUs

Even though the number of vehicular nodes can vary in a real-world scenario based on the amount of incoming and outgoing traffic, the number of RSUs in a given geographic area is typically a constant, unless some of the RSUs malfunction or new RSUs are installed. Therefore, for a given simulation run, we set the number of RSUs to a constant. However, we also vary the number of RSUs in a given geographical area (from 1 to 64), just like the number of vehicular nodes varied among different simulation runs when we test the effectiveness of the proposed optimization model under different network sizes. We configure the RSU network as a wired network as it represents I2I communication which is typically installed as a wired network [42]. We divide the vehicular network map into a rectangular grid and place RSUs at fixed horizontal and vertical spacing. This horizontal spacing is fixed (δx=130 m), and vertical spacing (δy) between two consecutive RSUs is calculated based on the dimensions of the map and the number of RSUs installed. Equation (25) shows the calculation of the vertical spacing.

(25) $$\begin{array}{c} \delta \;y=\frac{W}{Ceil(N_{RSU}/N_{row})}\;;if\;N_{RSU}>0 \end{array}$$

**Figure 6:**
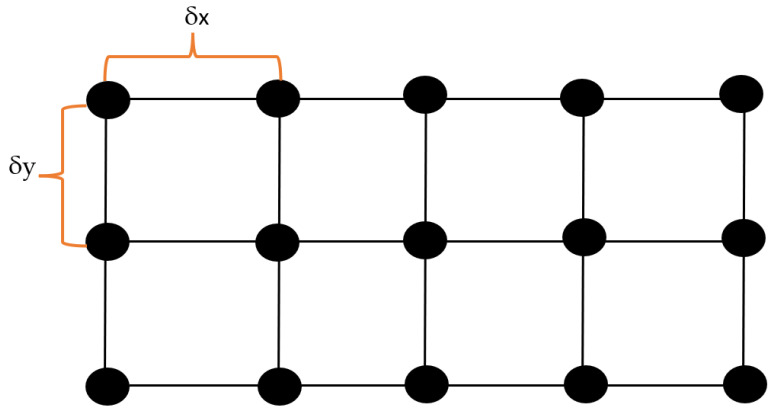
The placement and the connections of the RSUs in a given map.

In Equation (25), W is the width of the map and $N_{RSU}$ is the number of RSU nodes, $N_{row}$ is the number of RSU nodes per row. Figure 6 shows the placement of RSUs in a given map with interconnections between the RSUs.

Note that as evident from Figure 6, the RSU network is a mesh network having point-to-point links connecting with neighbor RSUs. Furthermore, there is a common bus for communication between a given RSU and SDN controller server, so that it has to use Carrier Sense Multiple Access-Collision Detection (CSMA-CD) for such communication. The data rate of the wired backbone (point-to-point links and CSMA-CD bus) is set to match Gigabit Ethernet parameters. Accordingly, the data rate is set as 1000 Mbps and the propagation delay is set as 10 μs. Furthermore, all RSUs are equipped with a DSRC interface for I2V and V2I communication. The CSMA-CD channel is connected to the controller in the centralized and hybrid SDVN architectures.

#### 4.2.5. Data and Metadata Packets

We collect the status data ($L_{st}$), namely the node Identifier (ID), timestamp, three-dimensional position, velocity, and acceleration of nodes in the vehicular network. These status data are collected by the control plane to make network decisions. We create a packet tag to embed the status data into a User Datagram Protocol (UDP) packet. Due to the use of real traffic data for vehicular nodes using a TCL file in Network Simulator 2 (NS2) mobility model, vehicular acceleration cannot be directly obtained from the mobility model. However, the position and velocity of vehicular nodes at a given instant of time can be obtained directly from the mobility model. Using the velocities and elapsed time between two positions (100 ms unless otherwise specified), we manually calculate the acceleration of a given node at runtime.

**Data packet:** For the hybrid architecture, we embed the set of one-hop neighbors ($S_{i}$) in the broadcasting data packet to reduce the transmission overhead. Thus, we consider the total broadcasting payload size in distributed and centralized architectures containing status information as 84 bytes and the broadcasting payload size of the hybrid architecture as $84+4N_{i}$. Thus, the total broadcasting encapsulated data packet size is 204 bytes for the DSRC communication channel (status data broadcasting takes place only in the DSRC channel) for centralized and distributed architectures. The total broadcasting encapsulated packet size of the hybrid architecture is $204+4N_{i}$ in the DSRC communication channel. Note that the payload sizes of the packets unicast to the control server by the agents differ based on the number of neighbors broadcasting to the agent/node in the hybrid and centralized architectures. In a hybrid architecture, a set of $S_{i}$ of the neighbors is also transmitted in addition to the status data payload. Therefore, the unicasting payload size of an agent in a hybrid architecture is at most $4\sum_{m\in S_{i}}\left(N_{m}Z_{m}+1\right)+84\left(N_{i}+1\right)$. In hybrid architecture, we use an adaptive approach where $S_{m}$ of broadcasting nodes will be sent by an agent only if it changes compared to the previous data-gathering cycle. The unicasting payload size of a node in centralized architecture is 84($N_{i}+1)$. Thus, the total unicasting data payload size of an agent in a hybrid architecture is at most $92+4\sum_{m\in S_{i}}\left(N_{m}Z_{m}+1\right)+84\left(N_{i}+1\right)$ and $110+4\sum_{m\in S_{i}}\left(N_{m}Z_{m}+1\right)+84\left(N_{i}+1\right)$ for Ethernet and LTE communication channels, respectively. Furthermore, the total unicasting data payload size of a node in the centralized architecture is $92+84(N_{i}+1)$ and $110+84(N_{i}+1)$ for Ethernet and LTE communication channels, respectively.

**Metadata packet:** In order to solve the data collection problem for optimization, metadata should be collected by the controller. As specified in the methodology section, there is a broadcasting metadata packet and a unicasting metadata packet. In the first data-gathering cycle, all nodes broadcast the metadata, and in subsequent data-gathering cycles, only the agent nodes broadcast metadata in the hybrid architecture. The broadcasting metadata packet consists of the node ID and timestamp information only, thus its payload size is limited to 12 bytes. However, the total size of a broadcasting metadata packet for DSRC is 132 bytes and 104 bytes for Ethernet. The broadcasting frequency is equal to the data collection frequency (*f*), which we vary to evaluate the performance of the solution. When broadcasting metadata is received at a given node, if the sender node already exists in the neighbor list, its timestamp will be updated. Otherwise, if the node does not exist in the neighbor list, it will be added as a new node with a new timestamp value. After adding the neighbor, the received node will also do a refreshing operation where old neighbor nodes (neighbor nodes whose timestamp value is greater than a threshold value) will be removed from the neighbor list since a recent broadcast from those old nodes has not been received. The unicasting uplink metadata packet of an $i^{th}$ node, which is sent only in the very first data-gathering cycle, consists of node ID of $i^{th}$ node, $S_{i}$, and current timestamp value. The payload size of unicasting uplink metadata is given by $12+4N_{i}$. Thus, the unicasting uplink metadata total packet size is $122+4N_{i}$ and $104+4N_{i}$ for LTE and Ethernet communication channels, respectively. The controller will compute the $B_{i}$ and $N_{i}$ values upon receiving a unicast uplink metadata packet by a node. Broadcasting downlink metadata received by a given RSU or vehicular node consists of the full solution of the optimization, which are the values for *z* and *x* decision variables of the optimization problem provided by the optimizer at the controller. A given node should extract the *z* and *x* values corresponding to the given node from the full solution received using the node ID. Thus, the payload size of broadcasting downlink metadata is just 2*N* bytes and the total packet size is 110 + 2*N* bytes and 94 + 2*N* bytes for LTE and Ethernet communication channels, respectively.

#### 4.2.6. Controller Node

The controller node is placed in a fixed location in a given map at simulation runtime, as it can occur in a real-world scenario. This node is equipped with a CSMA net device to directly communicate with the RSUs and a point-to-point wired connection between PGW and the controller node to communicate with the vehicular nodes through LTE.

#### 4.2.7. Configuration of the Data Collection Optimization Model

We use a Gurobi commercial optimizer to solve the data collection optimization. We use the Gurobipy python library to solve the problem of optimization of data collection by calling a python script for solving the optimization from NS3 script at run time to provide a solution based on the metadata collected by the controller. The metadata collected by the controller is saved in a Comma Separated Values (CSV) file to be read by the python script as input to the optimization model. Similarly, once a solution is found by the optimizer, it will be saved in another CSV file to be read by the controller in the NS3 environment. In a simulation run, we call the optimization solver at the rate of nominal optimization frequency ($f^{\prime }$) if and only if the entropy change in the network is greater than a threshold entropy change, compared to the last time the network was optimized. In this research, we set k=1 in Equation (12) such that the nominal optimization frequency ($f^{\prime }$) is the same as the data collection frequency (*f*). We set the communication cost per byte from the $i^{th}$ node to neighbor *m* ($C_{im}$) as 1 for wired communication, and 2 for DSRC communication [56]. The communication cost per byte for an agent ($C_{i}$) is set to the highest value of 40, as it involves cellular communication, which is usually 20 times the cost per byte of DSRC communication [57]. In order for the broadcasting delay from the $i^{th}$ node to the $m^{th}$ neighbor ($D_{im}$) comparable with the set communication cost per byte values, we calculate all delay parameters in milliseconds. The agent nodes’ combined communication ($B_{i}$) is input to the objective function in Equation (11) in milliseconds (typical values lie in the range 2 to 60). Therefore, to give equal priority for agents combined communication cost and overhead minimization (term 2 and term 3 in the objective function in Equation (11)) compared with the broadcasting nodes cost minimization (term 1 in the objective function whose typical values lie in range 1 to 30), $C_{CN}$ is set to two and both $C_{CA}$ and $C_{O}$ are set to one.

For a maximum of 256 nodes in the vehicular network, the solution time for solving the IQP model using the Gurobi IQP solver on average is only 40 ms. Thus, for very large-scale networks, it can be solved in parallel such that the solution time will not be much larger. Therefore, a heuristic solution mechanism for this problem is not needed in this context.

We summarize the main simulation parameters as shown in Table 2.

### 4.3. Results on Optimization of Data Collection

Distributed architecture has only the broadcasting step in data collection and does not have a data unicasting step to the control server. However, in order for future researchers to investigate more in this area, we perform a comparative analysis of the proposed optimization framework for the hybrid architecture against the distributed SDVN architecture as well. Furthermore, we compare it against the centralized architecture, which indeed has the unicasting communication with the centralized control plane in addition to the broadcasting step, which is a fair comparison against the proposed method, as both methods have unicasting and broadcasting steps.

#### 4.3.1. Effect of Network Entropy Change Threshold for Average Communication Cost, Average Channel Utilization, Average End-to-End Latency, Packet Delivery Ratio, and Optimization Percentage

**Figure 7:**
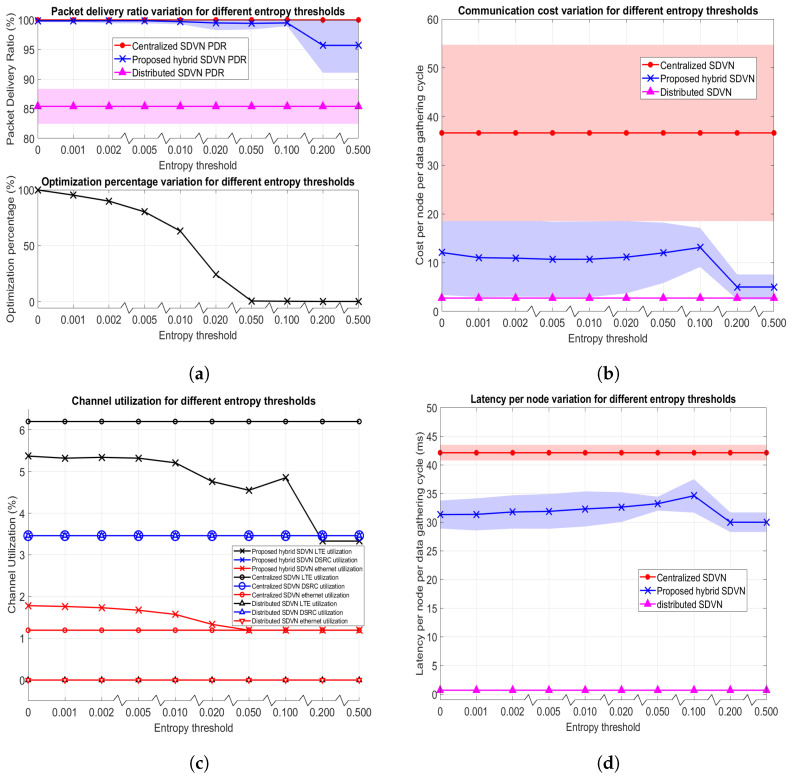
Performance evaluation of the data collection methods under different entropy threshold values. (**a**) Packet delivery ratio and optimization percentage variation for different entropy thresholds. (**b**) Communication cost per node per data-gathering cycle variation for different entropy thresholds; (**c**) Average channel utilization per data-gathering cycle variation for different entropy thresholds. (**d**) End-to-end latency per node per data-gathering cycle variation for different entropy thresholds.

In this experiment, the urban mobility scenario given in Figure 5a with the maximum speed for vehicles set to 60 kph is employed where both the data collection frequency (*f*) and nominal optimization frequency ($f^{\prime }$) are set to 2 Hz. In this scenario, 120 vehicles, and 60 RSUs exist in the network where the variable for the proposed data collection in a hybrid SDVN architecture is the network entropy threshold (T). There exists no variable for the centralized SDVN architecture and distributed SDVN architecture in this experiment. Performance evaluation metrics are assessed by varying the T value and the results for the data collection in three architectures are as shown in Figure 7. Note that colored areas in the graphs indicate the 95% Confidence Intervals (CIs).

The motivation to check an entropy threshold value to decide on optimization is to reduce the communication utilization and costs that occur in the optimization step, such as downlink broadcasting of the solution. Thus, in this approach, for minor changes in the network, finding a new solution for optimization will not be carried out. However, for significant topology changes, a new solution to the optimization is found out.

It is clear from the PDR result in Figure 7a that for entropy thresholds greater than or equal to 0.005, the average packet delivery ratio is less than 99.7%. Thus, at a 2 Hz nominal optimization frequency, only entropy thresholds less than or equal to 0.005 are reliable enough to deliver packets at the destination. A benchmark PDR of 99.9% is required for high-end V2X services [48,58]. However, at a 2 Hz optimization frequency, the proposed method fails to achieve the 99.9% PDR benchmark required for vehicular communication by 0.02%. However, as evident in Section 4.3.2, the proposed method will achieve the reliability benchmark of 99.9% at higher frequencies (frequencies greater than or equal to 4 Hz). The aim of this experiment is to study the effect of entropy threshold on reliability, cost, latency, etc., at a fixed frequency of 2 Hz. On the other hand, at a 2 Hz data collection frequency, the PDR of the centralized architecture has always been 100%. This high reliability in the centralized architecture is due to the fact that a given packet originating at a node is transmitted to the centralized controller by the node itself and all neighbors of the nodes that receive the packet. Thus, redundant data is transmitted by multiple nodes, however, it ensures that a given packet is always received at the centralized controller from one or more nodes. On the other hand, the packet delivery ratio for data collection in distributed SDVN architecture is much poorer than both other architectures, as it has an average value of 85.4%. This poor packet delivery ratio in VANETs occurs due to the presence of isolated nodes in the vehicular network where a data packet broadcast is not delivered to any other node.

When considering the transmission cost, as evident from Figure 7b, the proposed optimization for SDVN architecture has a much lower cost at all entropy threshold values compared to that of the centralized architecture. The average cost of the centralized architecture is more than three times that of the proposed method. The cost for the proposed method slowly reduces with an increment of entropy threshold up to entropy threshold 0.005, then gradually increases with the threshold value, and then suddenly drops to a lower value for thresholds greater than 0.10. At 0 entropy threshold, the cost is high due to the optimization percentage being 100% (always optimized), and excess resources are consumed to broadcast the optimization solution to the nodes. So, up to a threshold of 0.005, the cost reduces gradually as a higher threshold reduces the optimization percentage, as evident from Figure 7a. After 0.005, the cost begins to gradually increase as an optimization solution that minimizes the cost is not found due to the higher threshold values even when the topology changes. However, the smallest communication cost exists for distributed SDVN architecture, as evident from Figure 7b as the VANETs only have DSRC communication cost and do not involve communication with the centralized planes.

When considering the channel utilization results in Figure 7c, all proposed optimization for hybrid architecture, distributed, and centralized SDVN architectures have exactly the same DSRC utilization for any entropy threshold value. However, LTE utilization and Ethernet utilization differ among the three architectures. The proposed method has a higher Ethernet utilization for entropy threshold values less than 0.050 and Ethernet utilization for the proposed method approaches those of the centralized architecture for entropy threshold values greater than 0.050. Furthermore, the proposed method always has a lower LTE channel utilization for any entropy threshold value than that of the centralized architecture. The highest LTE utilization for the proposed method can be observed for the entropy threshold of zero and a decreasing trend can be observed with the increment of the entropy threshold. At low entropy threshold values, optimization is done frequently so that the LTE channel for downlink broadcasting of the solution is frequently utilized. On the other hand, for the distributed SDVN architecture, both LTE communication channel and Ethernet communication channel utilization is always zero as those channels are not utilized by VANETs.

Now, let us consider the result on end-to-end latency in Figure 7d. As evident from Figure 7d, the proposed optimization for hybrid architecture has a much lower latency than the centralized architecture for any entropy threshold value, as the upper limit of the 95% confidence interval of the proposed method’s latency lies much lower than the lower limit of the 95% confidence interval of the centralized architecture’s latency values. An increasing trend for the latency can be observed with the increment of the entropy threshold values for the proposed method, except after a threshold of 0.10, where there is a sudden drop in latency. On the other hand, the average latency for VANETs is much lower than both the proposed method and data collection for centralized architecture, as VANETs do not utilize LTE and Ethernet communication channels.

When analyzing the overall result, there are sudden drops in PDR, communication cost, LTE channel utilization, and latency, as evident from Figure 7a–c and Figure 7d, respectively, in the proposed data collection method for hybrid SDVN architecture for entropy thresholds greater than 0.10. This sudden decrease in cost, latency, LTE utilization, and PDR is due to the fact that for entropy thresholds greater than 0.10, optimization has occurred only in the very first data-gathering cycle, and a new solution has not been found after that, as proven by the optimization percentage values (0.8%) in Figure 7a. Therefore, PDR drops as broadcasting nodes become isolated and a new solution for optimization is not found due to the large entropy threshold. Thus, due to poor PDR, the decrement in cost, LTE utilization, and latency for higher entropy thresholds greater than 0.10 cannot be interpreted as performance gains.

Now, let us determine an optimum value for the threshold. As entropy thresholds less than or equal to 0.005 resulted in a PDR of 99.7%, the optimum value should be selected from that set. In terms of cost and channel utilization, the optimum threshold value is 0.005, as evident from Figure 7b,c, and in terms of end-to-end latency, the optimum threshold is 0.000 as evident from Figure 7d. At a threshold of 0.000, optimization always takes place, and at a threshold of 0.005, optimization takes place 80% of the time, as evident from Figure 7a. We expect the reduction in cost for a threshold of 0.005 than that of 0.000 is due to this 20% reduction in optimization, which requires extra resources for the downlink broadcasting step. Thus, considering the advantage in channel utilization, and cost, and as it has the highest average PDR of 99.7% for the considered nominal optimization frequency of 2 Hz, we set the optimization threshold to 0.005 for the rest of this paper unless otherwise specified.

#### 4.3.2. Effect of Nominal Optimization Frequency ($f^{\prime }$) for Average Communication Cost, Average Channel Utilization, Average End-to-End Latency, and Average Packet Delivery Ratio

**Figure 8:**
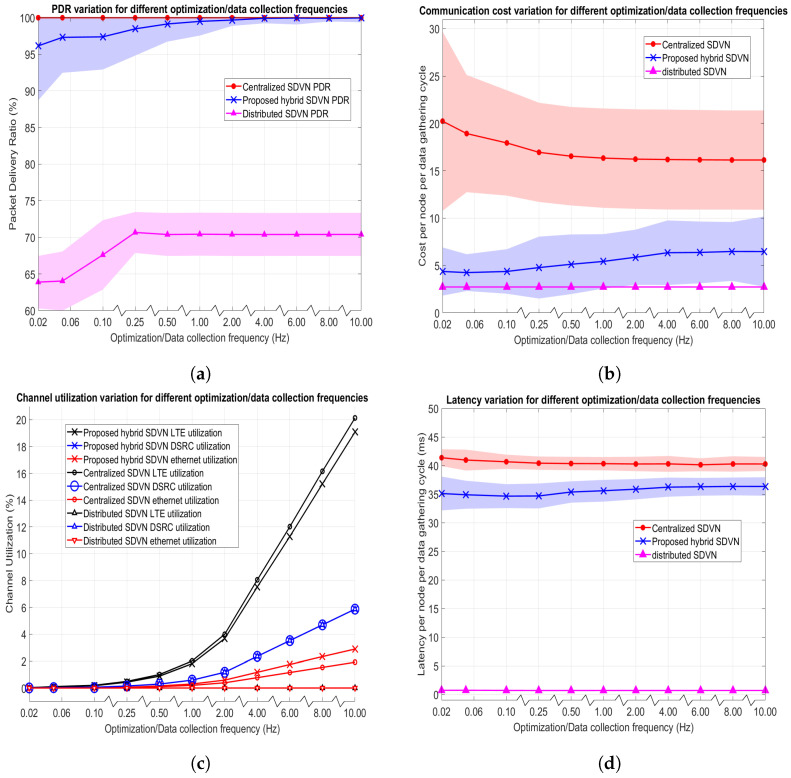
Performance evaluation of the data collection methods under different optimization/data collection frequencies. (**a**) Packet delivery ratio variation for different optimization/data collection frequencies; (**b**) Communication cost per node per data-gathering cycle variation for different optimization/data collection frequencies; (**c**) Average channel utilization per data-gathering cycle variation for different optimization/data collection frequencies; (**d**) End to end latency per node per data-gathering cycle variation for different optimization/data collection frequencies.

In this experiment also, an urban mobility scenario with the maximum speed of vehicles set to 60 kph is selected where there are 40 vehicles and 20 RSUs in the simulation. The network entropy threshold value for optimization is set as zero as needed to study the effect of nominal optimization frequency in this experiment. Otherwise, if an entropy threshold is set, it will have an effect on the results for nominal optimization frequency. Thus, a new solution to the optimization is found at a rate of nominal optimization frequency. As $f=kf^{\prime }$, we set the data collection frequency (*f*) equal to the nominal optimization frequency ($f^{\prime }$) by setting *k* value to 1. *f* and $f^{\prime }$ which are synced together are the variables in this experiment for the proposed data collection in hybrid SDVN architecture, and *f* is the variable for the data collection in centralized and distributed SDVN architectures. The results obtained accordingly are as shown in Figure 8.

Let us consider the result concerning the packet delivery ratio in Figure 8a. For any data collection frequency (*f*), the average PDR of the centralized architecture always achieves a value of 100% due to redundant data transmission from multiple nodes in this architecture. On the other hand, for the proposed optimization for data collection, the average PDR is less than 99% when the optimization frequency is less than or equal to 0.5 Hz. For the proposed optimization, the PDR always tends to increase with the increment of the frequency, as evident from Figure 8a. The reason for an increment in PDR with frequency is due to the fact that with an increment of optimization frequency, the probability of occurrence of data loss (packets not being delivered) due to broadcasting nodes being isolated (problem shown in Figure 3a) is low. The proposed method achieves an average PDR of 99.7% at 2 Hz and achieves 99.9% for frequencies greater than or equal to 4 Hz. Thus, in order to achieve a vehicular communication reliability benchmark of 99.9%, the optimization frequency must be greater than or equal to 4 Hz. As suggested by the lower average PDR values and low confidence interval lower limits for PDR in Figure 8a, nominal optimization frequencies less than or equal to 0.5 Hz are not acceptable for vehicular communication using the proposed method. On the other hand, the data collection using distributed SDVN architecture has the lowest average packet delivery ratio at any data collection frequency. The PDR for the distributed SDVN architecture tends to increase for data collection frequencies in the range of 0.02 to 0.25, and then has been saturated at a fixed value for higher frequencies indicating that data collection frequency has a lower impact on PDR in VANETs.

As evident from Figure 8b, the centralized architecture’s cost per node per data-gathering cycle is always higher than that of the proposed optimization for the hybrid SDVN architecture for any given optimization/data collection frequency. While the centralized architecture’s cost gradually decreases with the increment of the data collection frequency (*f*), the cost of the proposed data collection for hybrid architecture gradually increases with the increment of the optimization frequency ($f^{\prime }$). Furthermore, the gap (cost) between the proposed method and data collection for centralized architecture reduces with the increment of the optimization frequency as evident from Figure 8b. On the other hand, the average cost per node per data-gathering cycle for the distributed SDVN architecture remains at a constant value of 2.734 and does not vary with the data collection frequency.

Now, let us analyze the result for channel utilization given in Figure 8c. It is clear from Figure 8c that for all data collection methods, the channel utilization for any given channel increases with the increment of optimization frequency (data collection frequency for centralized and distributed architectures). The DSRC utilization curves for all 3 methods overlap, suggesting that all methods have the same DSRC utilization for a given frequency. At all the frequencies, the proposed method’s LTE utilization is lower than that of the centralized architecture, while the Ethernet channel utilization of the proposed method is always higher than that for the data collection in a centralized architecture. That is because the proposed method tends to select more Ethernet channels as agents, which have lower communication costs, and tends to reduce cellular communication, which has a higher cost. Another important fact about channel utilization is that the channel utilization gap between the centralized and hybrid architectures for LTE and Ethernet communication channels increases with the increment of the nominal optimization frequency, as evident from Figure 8c. Thus, in terms of communication cost and channel utilization, 0.02 is the best frequency for the proposed method for data collection. On the other hand, the LTE and Ethernet channel utilization for the distributed SDVN are always zero for any frequency.

Now, let us consider the effect of optimization/data collection frequency for end-to-end latency. As evident from Figure 8d, the latency per node per data-gathering cycle for the data collection in a centralized architecture gradually decreases with the increment of the data collection frequency. On the other hand, for the data collection using the proposed method, the latency tends to decrease for nominal optimization frequencies from 0.02 to 0.25 and then begins to slowly increase with the increment of the frequency. Furthermore, for all frequencies, the proposed method’s average latency values are much lower than those of the centralized architecture, and the proposed method’s latency 95% confidence interval upper limit lies well below the 95% confidence interval lower limit latency of the centralized architecture, thus we can confidently say that the proposed method’s latency is lower than the centralized architecture for any frequency. However, the latency gap between the proposed method and the data collection for the centralized architecture decreases with the increment of the frequency. By contrast, for the distributed SDVN architecture, the average latency is very low (0.71 ms) and the value does not vary with frequency.

In order to decide on an optimum value for the nominal optimization frequency, we should examine its effect on all communication costs, channel utilization, latency, and reliability. As analyzed, only frequencies greater than or equal to 4 Hz achieve the PDR benchmark (99.9%) for vehicular communication. As all communication costs, channel utilization, and end-to-end latency increase with increment of frequency for frequencies greater than or equal to 4 Hz for the proposed method, as evident from Figure 8b–d, we can conclude that 4 Hz is the optimum frequency which achieves a PDR of 99.9% and at the same time has a minimum cost, channel utilization, and latency.

#### 4.3.3. Effect of Total Number of Nodes for Average Communication Cost, Average Channel Utilization, Average End-to-End Latency, and Packet Delivery Ratio

**Figure 9:**
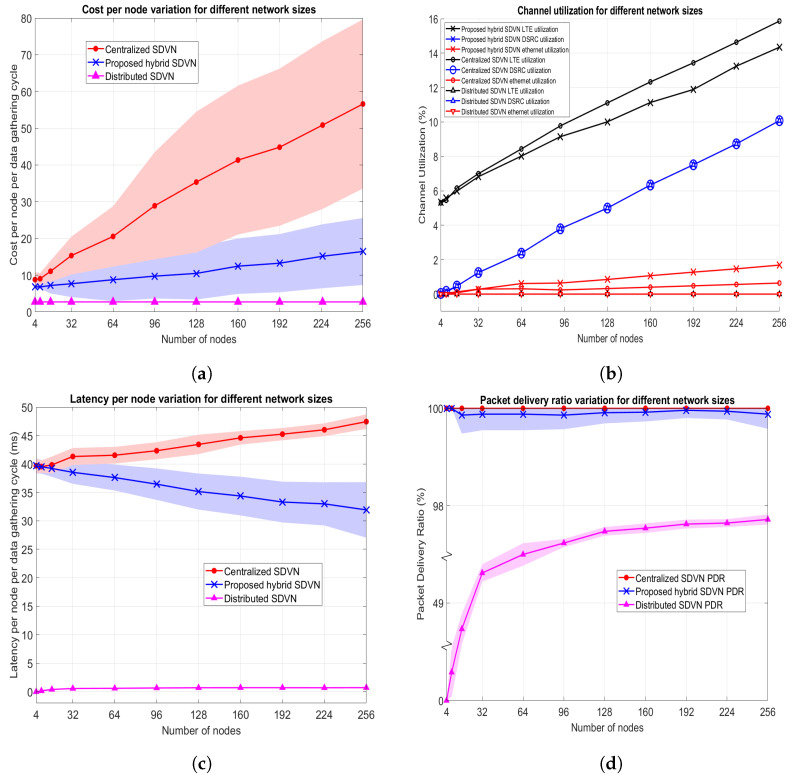
Performance evaluation of the data collection methods under different vehicular network sizes. (**a**) Communication cost per node per data-gathering cycle variation under different network sizes (**b**) Average channel utilization per data-gathering cycle variation for a different number of nodes. (**c**) End-to-end latency per node per data-gathering cycle variation for different network sizes. (**d**) Packet delivery ratio per data-gathering cycle variation for different network sizes.

In this experiment, we set both *f* and $f^{\prime }$ to 4 Hz as it was found in Section 4.3.2 that 4 Hz is the minimum optimization frequency to achieve a target packet delivery ratio of 99.9%. We simulate an urban mobility scenario having a 60 kph maximum vehicle speed. RSU to vehicular node ratio is maintained at 1:3, and the optimization threshold for proposed data collection in hybrid SDVN architecture is set as 0.005, based on the result obtained in Section 4.3.1. The variable for each architecture’s data collection is the total number of nodes in the vehicular network, which varies from 4 to 256. The performance of data collection methods was evaluated by varying the total number of nodes, and the results are shown in Figure 9.

It is very clear from Figure 9a that for both the proposed method in the hybrid architecture and for the data collection in the centralized architecture, the average cost per node per data-gathering cycle increases with the increment of the network size. The reason for the increment of average communication cost per node per data-gathering cycle for data collection in hybrid and centralized architectures with an increment of network size is due to the increment of average normalized network link-state entropy. As the nodes are placed in a fixed area of size 1500 m × 1500 m, the node density increases with the total number of nodes so that we can expect the average normalized network link entropy to increase. However, for the distributed architecture, the cost does not vary with network size, and the average cost is the lowest for the distributed architecture. For all network sizes, the average cost for the proposed method has always been less than that of the centralized architecture. Furthermore, the rate of increase of cost with network size is lower for the proposed method compared to data collection in a centralized architecture. For low network sizes, the cost reduction of the proposed method compared to the centralized architecture is lower, as evidenced by the lower difference between the cost average values, and the overlapping upper 95% confidence interval limit for cost in the proposed method with lower 95% confidence interval limit for the centralized architecture. However, with large networks, the proposed method significantly reduces the average cost per node per data-gathering cycle, as evidenced by the non-overlapping 95% confidence intervals of the cost for the proposed method and centralized architecture with a large difference between average values, as shown in Figure 9a. Thus, the proposed method will be very effective in reducing communication costs in large networks for data collection.

Now let us consider the channel utilization variation with respect to the total number of nodes in the network. As evident from Figure 9b, the channel utilization of all LTE, DSRC, and Ethernet channels for both the proposed method and data collection for centralized architecture increase with the network size. However, for the distributed architecture, the LTE and Ethernet communication channel utilization is always zero, irrespective of the network size. The DSRC channel utilization for all three architectures overlaps for all network sizes proving the equivalence of the data collection methods in DSRC communication channels. However, the utilization of LTE and Ethernet channels do not overlap for centralized and hybrid architectures. The rate of increment of LTE utilization with network size is lower, while the rate of increment of Ethernet utilization with network size is higher for the proposed data collection in hybrid SDVN architecture compared to data collection in centralized SDVN architecture. For small network sizes (less than or equal to 32), the utilization of LTE and Ethernet channels for the proposed method and centralized architecture is almost the same, as evident from Figure 9b. However, as the network gets bigger, the channel utilization gap between the proposed method and centralized architecture gets wider for both Ethernet and LTE communication channels. As the network gets bigger, the average LTE utilization of the proposed method varies less than that of the centralized architecture, and the average Ethernet utilization of the proposed method varies larger than that of the centralized architecture.

The result on latency given in Figure 9c clearly shows how the proposed method is effective in large networks to reduce the latency per node per data-gathering cycle. For small-size networks (size less than or equal to eight), the average latency and the 95% confidence intervals for the latency for both the proposed method and centralized architecture overlap, indicating that for very small networks, the latency of both methods is almost the same. As for this experiment, all nodes of the network exist in a fixed map of areas 1500 m × 1500 m, when the number of nodes increases, the node density increases, and thus, the average entropy of the network should also increase. Therefore, as the network gets more complex (bigger with higher average entropy), the latency for the centralized architecture increases as the number of links between the nodes increases. On the other hand, for the proposed method, when the network gets bigger and the average entropy of the network increases, the latency is reduced by the objective function of the proposed method, which is aware of the links between nodes and effectively forwards traffic through agents that have more neighbors, and by utilizing more Ethernet links as agents which have lower cost and delay. Therefore, for large networks, the proposed method will be very effective in reducing latency per node per data-gathering cycle. However, the latency per node per data-gathering cycle for the distributed architecture, which barely varies with the network size, is much lower than both the proposed method and the centralized architecture for any network size.

As evident from Figure 9d, a clear relationship between the network size and the PDR for the proposed method cannot be observed. For the data collection in centralized architecture, the PDR is always 100% for any network size due to redundant data transmission by the nodes. However, for network sizes of four and eight, the average PDR for the proposed method has reached 100%, and a minimum average value of 99.9% was observed for the network size of 16. For all other network sizes except 16, the average PDR of the proposed method had been more than 99.9% value, as evident from Figure 9d, which is a remarkable achievement, as it had attained the ultra-reliability benchmark required for vehicular communication. On the other hand, for the distributed SDVN architecture, the packet delivery ratio is extremely poor for lower network sizes, and it grows with the increment of network size, and the average PDR reaches 91% for network size of 256.

#### 4.3.4. Effect of Mobility for Average Communication Cost, Average Channel Utilization, Average End-to-End Latency, and Average Packet Delivery Ratio

**Figure 10:**
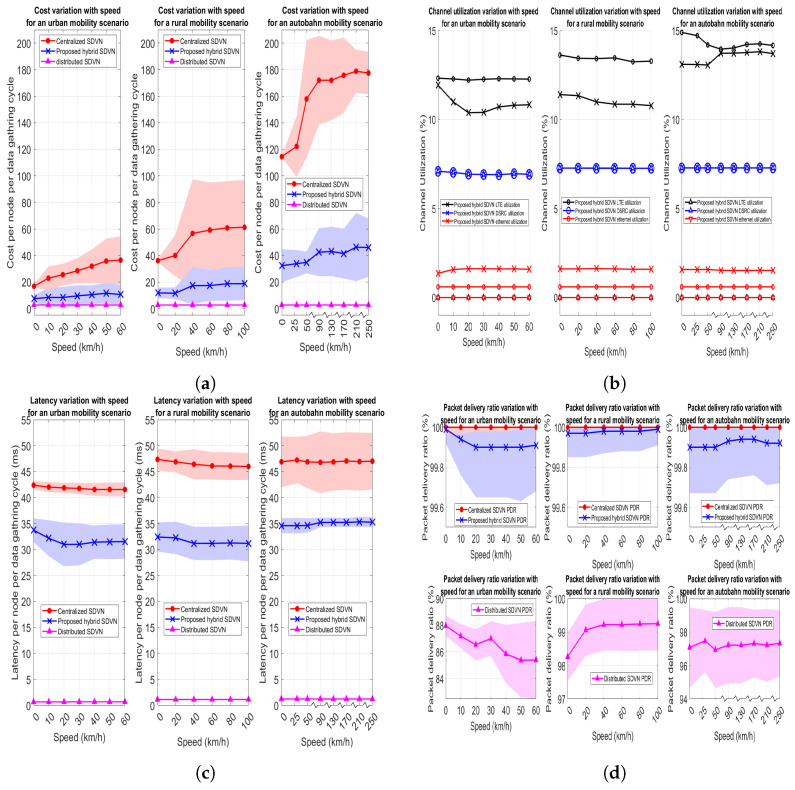
Performance evaluation of the data collection methods under different mobility scenarios. (**a**) Communication cost per node per data-gathering cycle variation for different mobility scenarios. (**b**) Average channel utilization per data-gathering cycle variation for different mobility scenarios. (**c**) End-to-end latency per node per data-gathering cycle variation for different mobility scenarios. (**d**) Packet delivery ratio per data-gathering cycle variation for different mobility scenarios.

In this experiment, 120 vehicles and 60 RSUs exist in the vehicular network where the data collection and nominal optimization frequencies are set to 4 Hz, based on the result in Section 4.3.2. The optimization threshold for the proposed data collection method for the hybrid SDVN architecture is set as 0.005, based on the results obtained in Section 4.3.1. The variable in this experiment is the allowable maximum speed of vehicles for three mobility scenarios: urban, non-urban, and autobahn. The proposed data collection method for the hybrid SDVN architecture, data collection in centralized SDVN architecture, and data collection in distributed SDVN architecture were evaluated for each of the mobility scenarios by varying the maximum allowable speed of vehicles to obtain the results in Figure 10.

It is very evident from Figure 10a that for all mobility scenarios, the average cost for data collection in both the proposed hybrid architecture and centralized architecture increases with speed. However, the costs for data collection for a given architecture for a given speed vary between different mobility scenarios for the hybrid and centralized SDVN architectures. The increasing order of cost for both architectures is urban, rural, and autobahn. The preceding order of cost can be explained due to the coverage of 139 m, 278 m, and 347 m for urban, rural, and autobahn mobility scenarios, respectively. Due to the preceding coverage specified, the average number of neighbors for a given node can be expected to vary in the same order. Thus, the average cost of the three mobility scenarios varies in the same order. In contrast, the cost for the distributed SDVN architecture does not vary with mobility and resides at a low fixed value of 2.734 irrespective of mobility. Another important fact to note in Figure 10a is that the gradients of the cost variation of the centralized architecture for all mobility scenarios are higher than the gradients of the cost variation for the proposed method. In other words, the increment of cost with the speed of the proposed method is lower compared to the centralized architecture. For all mobility scenarios, the average cost of the centralized architecture has always been higher than the average cost of the proposed method for all speeds. However, the performance gap for cost per node per data-gathering cycle between the two data collection methods varies in the increasing order as urban, rural, and autobahn.

Now, let us analyze the channel utilization variation shown in Figure 10b. For all mobility scenarios, the proposed method’s channel utilization for LTE has always been lower than that of the centralized architecture. In contrast, for all mobility scenarios, the proposed method’s channel utilization for Ethernet has always been higher than that of the centralized architecture. However, for all mobility scenarios, the DSRC utilizations for all three architectures overlap one another and have essentially remained the same with changes in the maximum allowable speed of vehicles. For a given speed, the channel utilization for DSRC and Ethernet between different mobility scenarios are almost the same, while significant differences can be observed in the LTE channel utilization between different mobility scenarios for the hybrid and centralized architectures. For the centralized architecture, the LTE channel utilization for each mobility scenario in the increasing order is urban, rural, and autobahn. This variation explains the reason for the communication cost variation for the centralized architecture with mobility in the exact same order. However, for the proposed method, the LTE channel utilization for the urban mobility scenario decreases from 0–20 kph, and then begins to increase from 30–60 kph. For the proposed method, the LTE channel utilization gradually drops with an increment of speed for the rural mobility scenario while it essentially remains the same for 0–50 kph, and then increases at 90 kph and again essentially remains the same from 90–250 kph in the autobahn mobility scenario. For the autobahn mobility scenario, the LTE channel utilization gap between the proposed method and centralized architecture gets narrower with the increment of the speed of vehicles. However, note that for the proposed method, LTE channel utilization values for the urban and rural mobility scenarios are similar, and relatively higher values can be observed for the autobahn scenario. The preceding pattern is the reason for observing similar communication cost values for urban and rural mobility scenarios, and relatively higher communication cost values for autobahn mobility scenarios for data collection using the proposed method. One of the main reasons for the increment of LTE utilization and cost for the autobahn scenario is that vehicles tend to exist in clusters in the autobahn mobility scenario, having a higher number of neighbors per node than in other mobility scenarios. On the other hand, LTE and Ethernet channel utilization for the distributed architecture is always zero for any speed under any mobility scenario, as those channels are not utilized in distributed architecture.

As it is shown in Figure 10c, the latency for both the proposed method and the centralized architecture tends to decrease with the increment of the vehicular speed for both urban and rural mobility scenarios. However, for the autobahn mobility scenario, the latency for the proposed method essentially remains the same for the 0–50 kph and 90–250 kph ranges, where a slight increment can be observed in the transition from 50–90 kph. For the autobahn mobility scenario, the latency remains almost the same with variation in speed except at 0–50 kph. For all mobility scenarios for both data collection methods in hybrid and centralized architectures, the change in latency with vehicular speed is quite a bit lower compared to the change in cost, which is much higher. The highest latency gap between the data collection methods for hybrid and centralized architectures is for the rural mobility scenario, where there is about a 15 ms latency gap between the proposed method and the centralized architecture. Both the urban mobility and autobahn mobility scenarios have similar latency gaps (about 12.5 ms) between the proposed method and the centralized architecture. Furthermore, for all mobility scenarios, at all vehicular speeds, the proposed method’s latency is much lower than the centralized architecture’s latency as the lower limit of the 95% confidence interval of the centralized architecture’s latency lies much above the upper 95% confidence interval limit of the proposed method, as evident from Figure 10c. However, for a given mobility scenario, the latency of the distributed SDVN architecture essentially does not vary with speed, and the latency values are much lower than those of both hybrid and centralized architectures.

When analyzing the result on the packet delivery ratio variation with mobility in Figure 10d, the PDR for the centralized architecture has always been 100% and has always been higher than the proposed method for all speeds in all mobility scenarios. However, the important thing to note is that the proposed method has always achieved the 99.9% average PDR value for all speeds in all mobility scenarios, which is the minimum PDR required for vehicular communication applications. For the proposed method, the best PDR has occurred for the rural mobility scenario, in which the PDR has always been more than 99.95% and there has been an increasing trend in PDR with the increment of the vehicular speed for the rural mobility scenario. On the other hand, for the proposed method in the urban mobility scenario, PDR has been close to 100% at zero mobility conditions and has gradually decreased to 99.9% by 20 kph and then remained at 99.9% for the remaining vehicular speeds (20–60 kph). For the autobahn mobility scenario, the PDR has remained at 99.9% for the proposed method for 0–50 kph, and for subsequent higher speeds, PDR is higher than 99.9%, as evident from Figure 10d. The packet delivery ratio for the distributed architecture is confidently less than both other architectures under all speeds for urban mobility scenarios, and the average value for rural and autobahn mobility scenarios is close to 99%. For the urban mobility scenario, a decreasing trend for PDR of distributed architecture can be observed with an increment of speed, whereas for the rural mobility scenario, an increasing trend can be observed, and in the autobahn scenario, a clear relationship cannot be observed.

The reason for the average communication cost per node per data-gathering cycle increment with mobility for data collection in centralized and hybrid architectures is that with high mobility, the probability for network topology change is high such that the number and type (vehicular or RSU) of neighbors of a given node can vary frequently. However, this increment in the probability of network topology change seems to have a lower impact on average channel utilization, average end-to-end latency, and packet delivery ratio for centralized and hybrid architectures. On the other hand, for the distributed architecture, latency and communication cost per node per data-gathering cycle essentially do not vary with mobility as for that architecture, the network topology does not have any impact on DSRC communication cost and latency per node per data-gathering cycle. However, as the average PDR of the distributed architecture depends on the average number of neighbors per node in a given instant, even though the variation is not clear, the PDR varies with mobility, as the average number of neighbors per node varies with mobility.

## 5. Discussion

For the hybrid SDVN architecture, we proposed a novel data collection method by modeling it as an integer quadratic programming problem. The decision for optimization is taken by inspecting the knowledge generated as normalized heterogeneous link-state entropy calculated using the collected neighbor set IDs and neighbor sizes. According to our best knowledge, we are the first to optimize data collection for the hybrid SDVN architecture, as it has not been previously investigated by any researcher. In order for future researchers to investigate, a performance evaluation for data collection was comparatively analyzed for the proposed data collection in the hybrid SDVN architecture, distributed SDVN architecture, and centralized SDVN architecture.

Vehicular communication supports a maximum latency of 500 ms for the transport of V2X messages between a piece of User Equipment (UE) supporting the V2X application and an RSU via another UE supporting the V2X application. Further, the BSM broadcast and similar uses of the cooperative awareness message for V2V safety generally allow a nominal 100 ms latency for human-assisted driving [59]. The 3GPP system should support a maximum latency of 3 ms, 10 ms, and 50 ms latency for message transfer among a group of UE pieces for fully autonomous driving within 200 m, 500 m, and 1000 m, respectively [48]. The requirement for low latency for vehicular network applications such as autonomous driving is highlighted in recent work [60]. It was evident from all research experiments in Section 4.3 that the proposed method’s latency per node per data-gathering cycle is confidently below 45 ms under any given scenario. Thus, the proposed data collection method’s latency lies below the maximum latency allowed (50 ms) by the 3GPP system for message transfer among a group of user equipment within 1000 m, and also it lies well below the 100 ms maximum latency allowed for status data collection (cooperative awareness message broadcasts such as BSM).

The reliability of vehicular communication is standardized based on the application. An ultra-reliability of 99.9% is required for the exchange of safety-related driving maneuvers [58]. For video data sharing applications, a minimum reliability of 90% is required, whereas, for human assistance systems, autonomous driving, and for machine-centric data analysis, a minimum reliability of 99.9% is required [48]. According to the results, a minimum nominal optimization frequency of 4 Hz and a maximum entropy threshold of 0.005 satisfy the minimum average packet delivery ratio (99.9% ultra-reliability benchmark) required for high-end vehicular network communication applications such as human assistance systems, autonomous driving, machine-centric data analysis, etc., as specified in the 3GPP standard for V2X applications. In the proposed data collection mechanism, the agent nodes collect and then send the collected data along with their own data, where packets can drop even when agent nodes collect data. However, the fact that the proposed method is ultra-reliable even under scenarios where packets can drop due to node isolation is a remarkable achievement. On the other hand, for the centralized architecture, the reliability is always almost 100% for any data collection frequency or any mobility scenario, or any network size. This high reliability occurs because of redundant data transmission by multiple nodes in a given neighborhood of a node. On the other hand, we observed that the PDR of distributed architecture is much poorer than both other architectures due to the presence of isolated nodes in the VANET. However, improved PDR for the distributed architecture was observed for rural and autobahn mobility scenarios and for large vehicular networks. The explanation for that increment is due to the higher coverage in rural and autobahn mobility scenarios and due to the increment of normalized network link entropy in large networks.

As the proposed data collection for hybrid architecture optimizes data collection by minimizing total communication cost and delay, as proven by the results, the communication cost and latency of the proposed method are confidently much lower than those of the data collection in the centralized architecture except for smaller networks (network sizes less than or equal to 16), as evident from Figure 9. The reason for the smaller performance gap between the two data collection methods in a smaller network is due to the tendency for nodes in the network to be located far apart from each other such that the normalized heterogeneous link-state entropy value of the network is close to zero. In such a case, all isolated nodes will be appointed as agents by the optimizer, and such a case is similar to the data collection in a centralized architecture. In other words, there is no difference in data collection by isolated nodes between the data collection for the hybrid and centralized architectures.

Let us now analyze the significance of the performance gap between the proposed method and the data collection in centralized SDVN architecture statistically. Let us consider the two null hypotheses listed below.

**H1.**  *The communication cost of the proposed method is higher than that of the centralized architecture;*

**H2.**  *The latency of the proposed method is higher than that of the centralized architecture;*

We consider a significance level of 0.05 in order to test the above-given hypotheses. If the probability *p* (how likely it is that data could have occurred under the null hypothesis) value is less than 0.05, the null hypothesis can be rejected, and the alternative hypothesis will be significant. As in all other tests (entropy test in Section 4.3.1, frequency test in Section 4.3.2, and mobility test in Section 4.3.4 except rural mobility scenario), it was proven that the communication cost and latency of the proposed method were confidently lower (as 95% upper CI of cost or latency of the proposed method lies below the 95% lower CI of the cost or latency of the data collection in centralized architecture) than that of the centralized architecture, we can conclude that both null Hypotheses H1 and H2 have a *p*-value of 0.00 such that alternative hypotheses are true and significant. However, we should examine the *p*-value for different network sizes, as evident from Section 4.3.3, and for different speed values for rural mobility scenarios, as evident from Section 4.3.4, where the performance gap between the data collection using the proposed method and that of the centralized architecture had been narrow, and 95% of the CIs overlap for some data points. Let us denote the *p*-value for Hypothesis H1 as *P*_H1_ and the *p*-value for Hypothesis H2 as *P*_H2_. We confirm that *P*_H1_ = *P*_H2_ = 0 for all speeds in the rural mobility scenarios, too. Therefore, alternative hypotheses are significant for all experimental results in Section 4.3.1, Section 4.3.2 and Section 4.3.4. Now, let us compute *p* values for the hypotheses for different network sizes, which are given in Table 3.

Thus, as evident from Table 3, the alternative hypothesis of H1, which is the cost of the proposed method is lower than that of the centralized architecture, is statistically significant for all network sizes, since the *P*_H1_ value is less than 0.05. However, the alternative hypothesis of H2, which is the latency of the proposed method is lower than that of the centralized architecture, is significant, since *P*_H2_ is less than 0.05, only for network sizes greater than or equal to 32.

The proposed method reduces the cost by using several strategies. The first one is that it reduces transmission overhead by collecting data using agents and transmitting the collected data by encapsulating it with a single header. Thus, when selecting agents, the proposed method selects agents that have more neighbors. The second one is by selecting agents that have lower communication costs and delays for the uplink. In a heterogeneous network, the optimizer tends to select RSUs as agents since they have lower communication costs and delays compared to vehicular nodes equipped with LTE links, which have comparatively higher communication costs and delays. Third, the proposed method adaptively sends metadata to the centralized planes, only if the metadata of an agent changes (only if the neighborhood of an agent changes). Fourth, it reduces downlink data transmission cost by performing optimization if and only if the normalized network link entropy change is greater than a specified entropy threshold. Thus, for large networks consisting considerable percentage of vehicular nodes, the cost for the proposed method is much lower than that for the data collection in the centralized architecture for any nominal optimization frequency or any mobility scenario.

The proposed method also reduces the average end-to-end latency using several methods. First, as agents, the optimizer selects nodes that have a lower delay. Thus, the optimizer tends to select RSU nodes as agents as they have a lower delay compared to vehicular nodes. For the proposed method, the data is sent to the centralized planes only by the agents. However, for the centralized architecture, as all nodes are agents, all nodes send data to the centralized planes, thus having a higher cost and delay than the proposed method even in a no RSU scenario. In a distributed architecture, both the cost and latency per node per data-gathering cycle is much lower than in both hybrid and centralized architectures. However, this low cost and latency are entirely because of the distributed SDVN architecture, or in other words, the VANET does not send data to the centralized planes where LTE and Ethernet communication channels are not utilized. Therefore, VANETs do not have knowledge regarding the entire vehicular network as there is no centralized controller collecting status or other data from nodes in order to make network decisions. Thus, the achievement of low delay and cost in VANETs is at the expense of not having centralized control, which gives programmability and flexibility to the network. On the other hand, the proposed data collection for the hybrid architecture has moderate latency and cost (higher than distributed architecture and lower than centralized architecture) values, at the same time ensuring centralized control in the SDN paradigm. Therefore, we can conclude that the proposed data collection is the method that achieves the lowest latency and cost out of the methods which have centralized control that get the advantages of programmability and flexibility.

In the process of achieving a lower average communication cost and latency by the proposed method, it is clear, according to the results, that the average LTE channel utilization of the proposed method has been utilized less, and the average Ethernet channel utilization has been utilized higher than that of the centralized architecture except for smaller networks (network sizes less than or equal to 16). The DSRC channel utilizations for all three methods overlap with one another for any mobility scenario, network size, or nominal optimization frequency. The reason for that is data broadcasting step in all methods behaves similarly such that DSRC channel utilization is the same. In other words, for the centralized and distributed architectures, all nodes broadcast data at a given time step. For the hybrid architecture, broadcasting nodes broadcast data and metadata while the remaining agent nodes broadcast metadata at a given time step such that the combined effect is equivalent to if all nodes transmit data. This is the reason for getting overlapping DSRC channel utilization for the data collection using all 3 methods.

The significance of this research is that the proposed optimization framework minimizes communication cost and latency when selecting broadcasting nodes and agents. The two problems—data loss due to node isolation and redundant data transmission by agent nodes which can occur if a new solution to the optimization is not found when the topology of the vehicular network changes—could be overcome with frequent optimization (at an optimization frequency greater than or equal to 4 Hz) and by setting a low (0.005) entropy threshold value, as evident from the results. As data is the basis for obtaining global network decisions such as routing optimization, network security, etc., at the centralized controller, data can be collected by the proposed optimization framework satisfying the ultra-reliability benchmark at the same time with lower cost and latency, in order to make accurate decisions at the controller without violating minimum latency requirements in vehicular communication.

## 6. Conclusions and Future Work

This paper presented an optimization framework for data collection in a hybrid SDVN architecture. For the hybrid SDVN architecture, we optimize data collection by modeling it as an integer quadratic programming problem. The results showed that the proposed optimization method has much lower communication cost and latency compared to the data collection for the centralized architecture for larger (well-connected) vehicular networks for any mobility scenario or for any optimization frequency. However, the proposed optimization should be done with a minimum optimization frequency of 4 Hz and a maximum heterogeneous network link entropy threshold of 0.005 in order to achieve the benchmark packet delivery ratio of 99.9% required for high-end vehicular network applications.

In future work, one can evaluate the performance of data collection when sensor (RGB camera, LIDAR sensors) data are collected. Furthermore, this research lays a foundation for applying machine learning to making network decisions in an SDVN. Thus, this research opens avenues for data scientists to optimally collect data at the centralized controller for applying machine learning to the collected data, which will improve the performance of the existing SDVN paradigm. Moreover, the proposed data collection mechanism for the SDVN paradigm can be extended to the Knowledge Defined Vehicular Network (KDVN) paradigm for data-centric network applications.

## Figures and Tables

**Table 1:** Summary of notations used in the proposed data collection optimization model.

Notation	Description
$x_{i},z_{i}$	Decision variable to represent the association of a node *i* for data collection as an agent, as a broadcasting node, respectively
$S_{i}$, S	Node ID set of all one-hop neighbors of node *i*, Set of one-hop neighbors of the whole network, respectively
$N,N_{i}$	Total number of nodes in the network, Total one-hop neighbors of node *i*, respectively
$B_{im},C_{im},D_{im}$	Combined communication cost, communication cost, and delay for communication between $i^{th}$ node and its $m^{th}$ neighbor, respectively
$B_{i},C_{i},D_{i}$	Combined communication cost, communication cost, and delay for communication from $i^{th}$ agent to controller, respectively
*f*, $f^{\prime }$	Update/Data collection frequency, optimization frequency, respectively
$L_{st,i},L_{se,i},L_{i}$	Status data payload, sensor data payload, and combined data payload of $i^{th}$ node, respectively
$p_{i},q_{i}$	Binary variable to represent the state of the collection of status data and sensor data from $i^{th}$ node, respectively
$C_{N},C_{DN},C_{DA},C_{CN},C_{CA},C_{LN},C_{O}$	Optimization coefficients
$H_{hom},H_{het}$	Homogeneous normalized network entropy, Heterogeneous normalized network entropy, respectively

**Table 2:** Summary table of simulation parameters.

Parameter	Value
Network simulation	NS-3.35
Optimizer	Gurobi 9.5.2
Plotting tool	MATLAB R2021a
Mobility scenario generation	SUMO version v1_14_1 and OpenStreetMap
Simulation time	600 s per each run
Maximum vehicles	200
Maximum RSUs	64
Maximum speed of vehicles	0–60 kph (Urban), 0–100 kph (Non-urban), 0–250 kph (autobahn)
Transmission protocol	User Datagram Protocol (UDP)
Communication channels	DSRC for (I2V, V2I, V2V), point to point between RSUs, CSMA from RSU to controller nodes, and LTE between vehicles and the controller node
Wi-Fi standard	IEEE 802.11p
DSRC transmission power	33 dBm (urban), 41 dBm (non-urban), 44 dBm (highway)
DSRC OFDM datarate	12 Mbps
DSRC propagation loss model	Cost-Hata (urban), 3-log distance (non-urban, autobahn)
DSRC propagation delay model	Constant speed propagation delay
DSRC error rate model	Nist error rate model
LTE pathloss model	Cost-Hata
LTE maximum transmit power	23 dBm
LTE SRS periodicity	2, 5, 10, 20, 40, 80, 160, 320 as given in Equation (24)
LTE fading model	Trace fading loss model
LTE EPC datarate	1000 Mbps
RSU backbone datarate	1000 Mbps
RSU backbone delay	10 μs
Broadcasting data payload size	84 bytes (centralized and distributed architectures), (84 + 4$N_{i}$) in hybrid architecture
Broadcasting metadata payload size	12 bytes (Exists in all nodes in the first data-gathering cycle and agent nodes in subsequent cycles in hybrid architecture)
Unicasting uplink data payload size	$4\sum_{m\in S_{i}}\left(N_{m}Z_{m}+1\right)+84\left(N_{i}+1\right)$ is the maximum probable payload size for hybrid architecture, 84($N_{i}+1)$ for centralized architecture, 0 for distributed architecture
Unicasting uplink metadata payload size	$12+4N_{i}$ (Exists only in the first data-gathering cycle of hybrid architecture)
Broadcasting downlink metadata payload size	2N bytes
Communication cost per byte	1—wired, 2—DSRC, 40—Cellular
Optimization coefficients	$C_{CN}=2,\;C_{CA}=1,\;C_{O}=1$
Data collection frequency	Variable (*f*) in the range [0.02, 10]
Nominal optimization frequency	Variable ($f^{\prime }$) in the range [0.02, 10]
Maximum velocity of vehicles	Variable ($V_{max}$) in the range [0, 250]
Number of Nodes	Variable (N) in range [4, 256]
Network link entropy change threshold	Variable (T) in range [0, 1]

**Table 3:** Table of *p* values of null hypotheses for different network sizes.

Network Size	*P*_H1_	*P*_H2_
4	0.0004	0.0559
8	0.0004	0.1358
16	0.0000	0.1249
32	0.0000	0.0021
64	0.0000	0.0004
{96, 128, 160, 192, 224, 256}	0.0000	0.0000

## Data Availability

Data and code are available for the journal to review. Data and code are available from the authors upon reasonable request after publication.
